# Ancient Sheep Genomes Reveal Four Millennia of North European Short-Tailed Sheep in the Baltic Sea Region

**DOI:** 10.1093/gbe/evae114

**Published:** 2024-05-25

**Authors:** Martin N A Larsson, Pedro Morell Miranda, Li Pan, Kıvılcım Başak Vural, Damla Kaptan, André Elias Rodrigues Soares, Hanna Kivikero, Juha Kantanen, Mehmet Somel, Füsun Özer, Anna M Johansson, Jan Storå, Torsten Günther

**Affiliations:** Human Evolution, Department of Organismal Biology, Uppsala University, Uppsala, Sweden; Human Evolution, Department of Organismal Biology, Uppsala University, Uppsala, Sweden; Human Evolution, Department of Organismal Biology, Uppsala University, Uppsala, Sweden; Department of Biological Sciences, Middle East Technical University, Ankara, Turkey; Department of Biological Sciences, Middle East Technical University, Ankara, Turkey; Human Evolution, Department of Organismal Biology, Uppsala University, Uppsala, Sweden; Department of Culture, University of Helsinki, Helsinki, Finland; Natural Resources Institute Finland, Jokioinen, Finland; Department of Biological Sciences, Middle East Technical University, Ankara, Turkey; Department of Anthropology, Hacettepe University, Ankara, Turkey; Department of Animal Biosciences, Swedish University of Agricultural Sciences, Uppsala, Sweden; Osteoarchaeological Research Laboratory, Stockholm University, Stockholm, Sweden; Human Evolution, Department of Organismal Biology, Uppsala University, Uppsala, Sweden

**Keywords:** sheep, ancient DNA, Baltic, breeds, domestication

## Abstract

Sheep are among the earliest domesticated livestock species, with a wide variety of breeds present today. However, it remains unclear how far back this diversity goes, with formal documentation only dating back a few centuries. North European short-tailed (NEST) breeds are often assumed to be among the oldest domestic sheep populations, even thought to represent relicts of the earliest sheep expansions during the Neolithic period reaching Scandinavia <6,000 years ago. This study sequenced the genomes (up to 11.6X) of five sheep remains from the Baltic islands of Gotland and Åland, dating from the Late Neolithic (∼4,100 cal BP) to historical times (∼1,600 CE). Our findings indicate that these ancient sheep largely possessed the genetic characteristics of modern NEST breeds, suggesting a substantial degree of long-term continuity of this sheep type in the Baltic Sea region. Despite the wide temporal spread, population genetic analyses show high levels of affinity between the ancient genomes and they also exhibit relatively high genetic diversity when compared to modern NEST breeds, implying a loss of diversity in most breeds during the last centuries associated with breed formation and recent bottlenecks. Our results shed light on the development of breeds in Northern Europe specifically as well as the development of genetic diversity in sheep breeds, and their expansion from the domestication center in general.

**Significance**

This study addresses the limited knowledge regarding the origins and diversity of sheep landraces and breeds, focusing on North European short-tailed (NEST) sheep in the Baltic Sea region. By analyzing ancient sheep remains from the Baltic islands, we found that these ancient sheep share a strong genetic similarity with present-day NEST breeds. Surprisingly, the ancient genomes displayed a relatively high genetic diversity compared to modern breeds, suggesting a loss of diversity over time. These results provide valuable insights into the development and genetic diversity of sheep breeds, contributing to conservation efforts and sustainable breeding practices in Northern Europe.

## Introduction

Sheep (*Ovis aries*) were domesticated in the Early Neolithic most likely somewhere in, or just to the east of, Anatolia (Zeder 2008). Sheep had spread into mainland Europe by around 8,000 BP with human farming populations expanding from that region during the Neolithic period. Sheep reached Scandinavia with the same expansion, probably following the Danubian route, by around 6,000 BP (Ryder 1984). The first Scandinavian farmers were associated with the Funnel Beaker culture (Fischer 2002; Sjögren et al. 2019) and spread as far north as central Sweden. The most common domestic fauna during this time period were pigs and cattle, followed by sheep but without strong indications of goat husbandry (Sjögren et al. 2023). The Funnel Beaker culture was succeeded, starting around 5,000 BP, by the Battle Axe Culture which was a local variation on the pan-European Corded Ware Complex (Malmer 2002). This time period saw a strong shift toward sheep husbandry (Malmer 2002; Larsson 2009). Studies of both Corded Ware and Battle Axe Culture-associated humans show that they have an ancestry component originating from the Pontic–Caspian steppe, associated with an expansion of human groups of the Yamnaya culture, which has not been observed in earlier individuals in Scandinavia (Allentoft et al. 2015; Haak et al. 2015; Malmström et al. 2019).

**Fig. 1.**
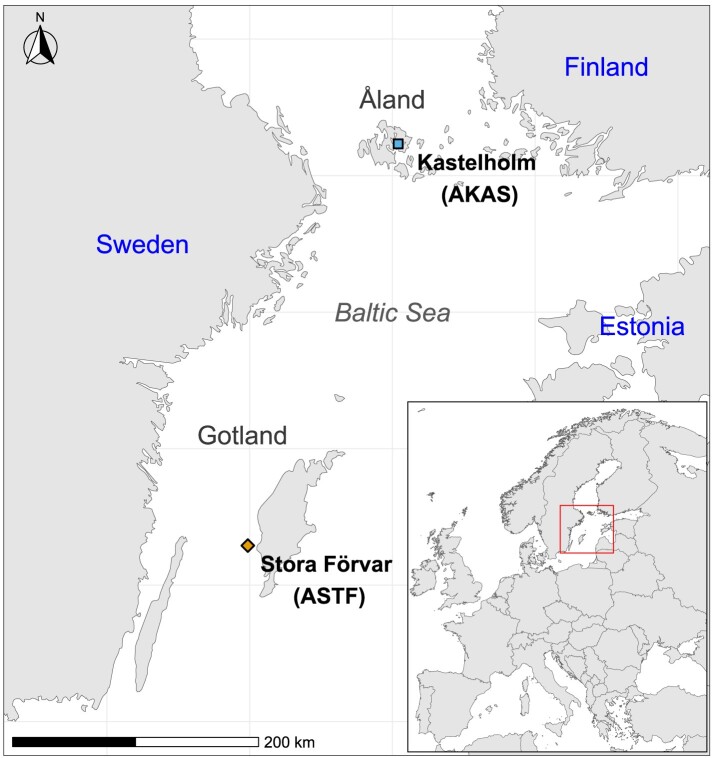
Map showing the Baltic Sea with some of the neighboring countries. Samples are marked by the square and the diamond. The inset map in the lower right corner shows the extent of the main map on a map of Europe. Map created using ggspatial (Dunnington 2022) using geographic data from the rnaturalearth packages (Massicotte and South 2022).

Today, northern Europe is home to the North European short-tailed (NEST) sheep, a group consisting of 34 breeds (Dýrmundsson and Niżnikowski 2010). These sheep are characterized by a short and tapering tail, which is often also covered in wool. With this short tail and dual-coated wool, the group is considered to be more “archaic” than other European sheep (as reviewed by Dýrmundsson and Niżnikowski 2010). Genetic studies have also found a unique pattern of retrovirus integrations making this group a potential relict of the first migrations of sheep into northern Europe (Chessa et al. 2009; Rannamäe et al. 2020). Although microsatellite studies found variation between different breeds (Tapio et al. 2005) they show comparatively low levels of genetic diversity (Lv et al. 2022), which can be attributed to some NEST breeds’ long breed history and the fact that many breeds have historical census sizes of only a few individuals. NEST are now found in an area stretching from Iceland to Russia and are believed to have spread from Scandinavia through Norse Viking expansion (Ryder 1983; Dýrmundsson and Niżnikowski 2010). They are closely related to other northwestern European breeds (Northern France, the British Isles, etc.), but most have small, isolated populations—both in mainland Scandinavia and Scotland and small islands like the Soay sheep of Saint Kilda (Dýrmundsson and Niżnikowski 2010; Stoffel et al. 2021). On the Baltic islands of Gotland and Åland, there are three local sheep breeds (see Fig. 1): The most common sheep on Gotland are of the breed “Gotland” (Swedish Board of Agriculture 2022) which was intentionally developed in the early 20th century as a commercial breed for their meat and pelts. Gute sheep, the traditional free-range breed on the island, went through a massive bottleneck in the mid-20th century as a result of the increase in popularity of the new Gotland sheep, but has recovered due to conservation efforts (Edberg 1986). Furthermore, the local sheep breed on the Åland archipelago is named after those islands (Tapio et al. 2005).

Ancient DNA (aDNA) has developed into a powerful tool for studying the spread and history of domestic animals (Frantz et al. 2020). Archeogenomic studies in sheep so far only include a few case studies in Anatolia (Yurtman et al. 2021), central Asia (Taylor et al. 2021), and Iran (Rossi et al. 2021). In northern Europe, aDNA studies have been restricted to uniparental markers (mitochondria and/or Y chromosomes; Niemi et al. 2013; Rannamäe, et al. 2016a, 2016b), which have limited power for understanding complex demographic processes due to their nonrecombining nature (Mourier et al. 2012) and are also sensitive to sex-biased processes. Nevertheless, these studies suggest a long continuity of sheep in the Baltic Sea region since at least the Late Bronze Age as indicated by similar uniparental haplotypes between ancient and today's sheep (Niemi et al. 2013; Rannamäe, et al. 2016a). Here, we generate ancient genomes of five Scandinavian sheep (up to 11.6X sequencing depth) dating to the Late Neolithic (on Gotland) and historical periods (on Åland). We investigate their relationship to modern NEST breeds and observe a surprising level of continuity over nearly 4,000 years. However, a recent reduction in genetic diversity seems to coincide with the development of separate breeds in the last centuries as well as with the replacement of local breeds with larger meat breeds such as Texel and Suffolk by many farmers.

## Results

We successfully obtained genomic aDNA from five sheep bones excavated from islands in the Baltic Sea—three from the cave Stora Förvar on Stora Karlsö, an island near Gotland, and two from the medieval Kastelholm Castle on Åland (Fig. 1), locations with potential connections to both sides of the Baltic Sea. While the history of the islands shows some parallels, there is no particular connection between the two archeological sites. Two samples from Stora Förvar (ASTF hereafter) were radiocarbon dated to the Late Neolithic around 4,000 cal BP while one sample from Kastelholm (AKAS hereafter) has been dated to historical times around the 16th century CE. The remaining samples that were not directly dated fit well within the same archeological context as the other samples, so we assume they are of similar ages. DNA preservation ranged from 1.3% (AKAS001) to 47% (ASTF002) endogenous DNA allowing us to shotgun sequence the genomes up to 11.6X autosomal coverage (Table 1). The well-preserved Late Neolithic material confirms Gotland and its limestone as an exceptional place for DNA preservation which has already facilitated several studies on human aDNA (Skoglund et al. 2014; Günther et al. 2018; Coutinho et al. 2020). The sequence data are showing fragmentation and deamination damage patterns as expected for aDNA (supplementary figs. S1 and S2, Supplementary Material online). Four individuals belonged to mitochondrial haplogroup B which is the most common haplogroup in European sheep while one individual (ASTF002) carried haplogroup A which is usually more common in modern Asian sheep breeds (Machová et al. 2022), but both haplogroups have been found in modern and ancient northern European sheep (Niemi et al. 2013; Rannamäe et al. 2016a, 2016b).

**Table 1:** Overview of samples sequenced for this study

Sample	Site	Autosomal coverage	Median read length (bp)	Sex	Age (2σ, 95.4% probability)	Mitochondrial haplotype
ASTF-001	Stora Förvar, Gotland	6.7153	49	Female	3957 to 3699 cal BP (Ua-71140)	B1a
ASTF-002	Stora Förvar, Gotland	11.6010	69	Male	4151 to 3936 cal BP (Ua-71139)	A1b
ASTF-003	Stora Förvar, Gotland	3.8368	49	Male	Late Neolithic^a^	B1a2a1
AKAS-001	Kastelholm, Åland	0.0703	64	Female	527 to 340 cal BP (Ua-71141)	B1a1
AKAS-002	Kastelholm, Åland	1.3350	48	Female	CE 1500 to 1550^a^	B1a2a

BP, before present (1950 CE).

^a^Contextual dates.

### Descriptive Population Genetic Analysis

**Fig. 2.**
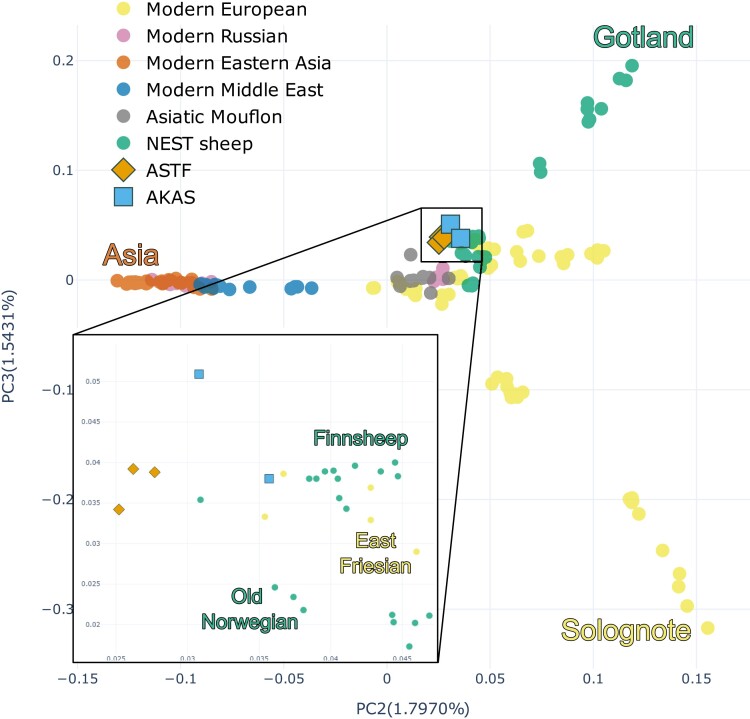
PCA biplot of the ancient Baltic sheep projected over the WGS dataset for PCs 2 and 3. PC2 matches the geographical distribution of modern sheep, while PC3 polarizes some of the NEST breeds from other European sheep. The inner square represents a zoom-in on the ancient samples.

To place the prehistoric and historic sheep samples into the broader genetic diversity of sheep, we merged them with large genomic datasets of modern-day sheep breeds from Eurasia and Africa. We first started with a dataset derived from whole genome resequencing data (WGS dataset hereafter) with single nucleotide polymorphisms (SNPs) that were ascertained in outgroups (other species of the genus *Ovis*; supplementary table S1, Supplementary Material online) to reduce the potential impact of ascertainment bias toward commercial breeds. The full dataset included about 2.7 million ascertained SNPs which were then called in 151 individuals from 31 Eurasian breeds as well as 10 Asiatic mouflons (*O. gmelini*) assumed to represent the wild ancestor of domestic sheep (supplementary table S2, Supplementary Material online; Naval-Sanchez et al. 2018; Deng et al. 2020; Li et al. 2020). We then projected the ancient sheep onto the major axes of variation (principal components, PCs) in the modern breeds. PC1 (explaining 3.69% of the variation) separates Asiatic mouflon from domestic sheep while further PCs sort out variation within domestic sheep (supplementary fig. S3, Supplementary Material online). PC2 (explaining 1.80% of the total variance) aligns breeds mostly along an East to West gradient while PC3 (1.54%) starts separating individual breeds forming a gradient between Solognote from France to Gotland from Sweden (Fig. 2). In this analysis, the ancient samples fall with European sheep, close to some NEST breeds (Finnsheep and OldNorwegian) and East Friesian but do not directly overlap with any of these modern breeds. This result is consistent with model-based clustering as implemented in ADMIXTURE (Alexander et al. 2009) where they are composed of similar ancestry components as modern NEST breeds but with some minor ancestry contribution from other groups (supplementary fig. S6, Supplementary Material online).

### Comparison to a Diverse Set of NEST Breeds

To further investigate the similarity of the ancient sheep to modern NEST breeds, we compiled a second dataset including more NEST breeds as well as breeds from Russia all genotyped on the Illumina Ovine Infinium HD 600K chip (Rochus et al. 2018, 2020a; Cao et al. 2021; Igoshin et al. 2022). The final dataset included 1,010 modern individuals and 484,428 SNPs for analysis (SNPCHP dataset hereafter, supplementary table S3, Supplementary Material online). Comparison with principal component analysis (PCA) and ADMIXTURE show similar results as for the WGS dataset, the ancient sheep display clear similarities to NEST breeds without directly matching any of them (supplementary figs. S4, S5, and S7, Supplementary Material online). This is particularly interesting as the SNPCHP dataset includes the two breeds from Gotland (Gotland and Gute) as well as the local sheep breed from Åland.

**Fig. 3.**
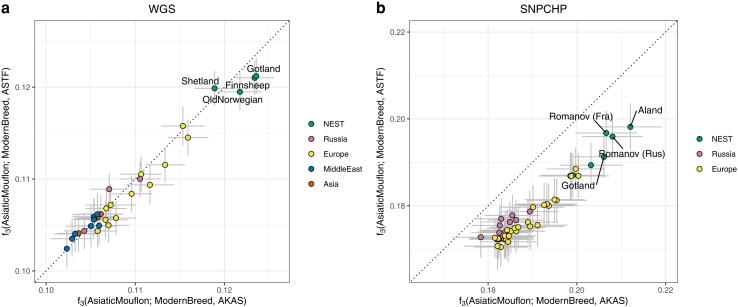
Outgroup *f*_3_ biplot measuring the shared drift of the ancient Baltic sheep from both sites/time periods versus modern breeds. Error bars show two block-jackknife standard errors. a) WGS dataset and b) SNPCHP dataset.

To identify the modern sheep breeds that share the most evolutionary history with the ancient sheep, we estimated shared drift using outgroup *f*_3_ statistics for both modern datasets. The estimates for ASTF and AKAS show a remarkably high correlation (Fig. 3) despite being from separate islands and nearly 4,000 years apart. For the WGS dataset (Fig. 3a), we see the highest amounts of genetic drift shared with the NEST breeds (Finnsheep and Gotland showing the highest values) and a substantial gap to other breeds including other European breeds. For the SNPCHP data (Fig. 3b), we see Åland sheep on top followed by Romanov and other NEST breeds. The inclusion of a more diverse set of NEST breeds as well as further breeds from Russia and Europe filled the gap between the top hits and all other breeds in this case. Finding Romanov sheep as one of the breeds with the highest amounts of shared drift with ancient Scandinavian sheep is remarkable as this Russian breed is assumed to have contributed to the gene pool of modern NEST breeds (Ghoreishifar et al. 2021). Notably, AKAS consistently displays more shared drift with modern European, NEST, and Russian breeds in the SNPCHP dataset (Fig. 3b) reflecting the larger shared evolutionary history compared to the chronologically earlier ASTF. This pattern is less pronounced in the WGS dataset although most European and NEST breeds show a similar tendency (Fig. 3a). Middle Eastern and Asian breeds were not part of the SNPCHP dataset, so this difference could be a combination of the composition of the datasets and the outgroup ascertained SNPs in the WGS dataset being less sensitive to very recent sheep history.

**Fig. 4.**
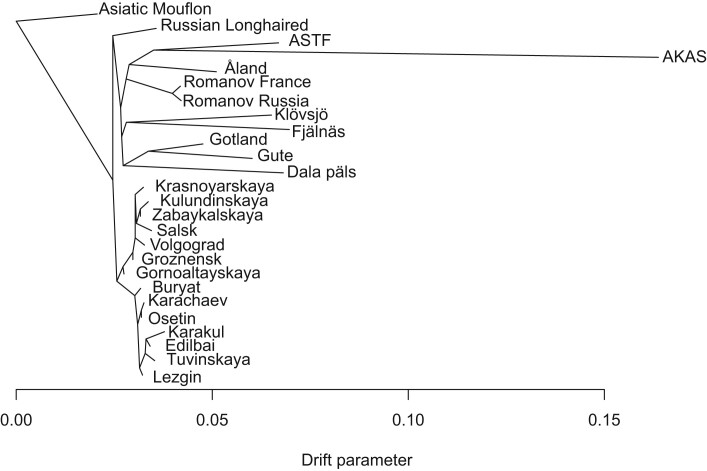
Maximum likelihood tree (using OrientAGraph; Molloy et al. 2021) based on the covariances of allele frequencies in the SNPCHP data restricted to NEST and Russian breeds. The tree was estimated with OrientAGraph not allowing any migration edges.

The outgroup *f*_3_ results can be corroborated by a maximum likelihood tree based on the allele frequencies using OrientAGraph, an extension of the popular TreeMix tool usually yielding better results with migration edges (Pickrell and Pritchard 2012; Molloy et al. 2021; Fig. 4, supplementary fig. S8, Supplementary Material online). In the OrientAGraph results, ASTF and AKAS group together with Åland as the closest group followed by Romanov who cluster with NEST breeds instead of with other Russian breeds. The private drift is much stronger in AKAS compared to ASTF which can be attributed to sample size and missing data, as they display the same amount of private drift when restricting the data to a single individual per group without missing data (supplementary fig. S9, Supplementary Material online). Residual matrices and results for up to three migration edges are shown in supplementary fig. S8, Supplementary Material online. The first migration edges are either between Russian breeds or between the ancients and the outgroup which could reflect an aDNA-related data quality issue. Consequently, we chose to display the tree for *m* = 0 in Fig. 4 as the migration events do not seem relevant to our main questions.

We complement the OrientAGraph analysis with a systematic exploration of admixture graph space using admixtools2 (Maier et al. 2023). To reduce running time, we excluded most Russian breeds (except Romanov, Russian Longhair, and Karakul) which also reduced the migration events involving Russian breeds. The first migration event involves 12% basal ancestry into a common ancestor of Karakul and the two ancient groups (supplementary fig. S10, Supplementary Material online) which can be considered somewhat consistent with the OrientAGraph results. Similarly, this could reflect aDNA-specific issues driving this outgroup attraction preventing us from interpreting any admixture results in this analysis.

While these results identify certain NEST breeds as closest modern-day relatives to ASTF and AKAS, they cannot be used as evidence for them representing the direct ancestor of any modern breed. An explicit test for population continuity (Schraiber 2018) rejected continuity for all breeds included in both datasets (supplementary tables S4 and S5, Supplementary Material online), while supporting continuity between our two ancient populations (supplementary table S4, Supplementary Material online). This result, however, may be explained by a lack of statistical power to reject continuity, as this method expects the reference “modern” (or temporally later) population to be of higher quality and sufficient sample size, requirements that are not met with the AKAS population. It also needs to be noted that this test is considered extremely strict and even minor levels of independent evolution or gene flow can lead to a rejection of the continuity model when data quality is sufficient (Schraiber 2018). In agreement with the OrientAGraph results (Fig. 4), models of discontinuity between modern and ancient samples estimate a substantial degree of private drift in the ancient populations which might be attributed to a combined effect of them representing island populations and aDNA data quality. Among all breeds, we also see that Romanov, Åland sheep, and other NEST breeds show the lowest levels of divergence, consistent with the results of the outgroup *f*_3_ and OrientAGraph analyses (supplementary tables S4 and S5, Supplementary Material online).

### Development of Genetic Diversity Over Time

**Fig. 5.**
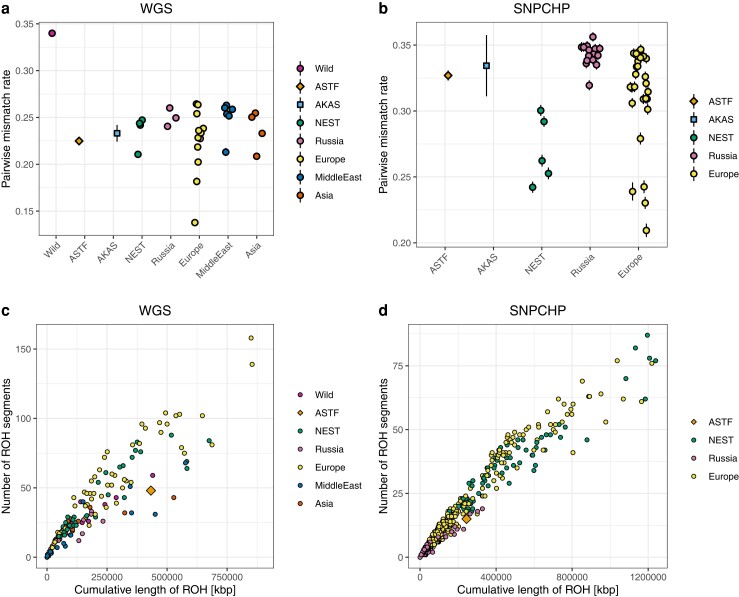
Genetic diversity results. Pairwise mismatch rate between randomly sampled individuals from the same group are shown in a) for the WGS dataset and b) for the SNPCHP dataset. Error bars indicate two block-jackknife standard errors (for most groups, the error bars disappear behind the symbols). This analysis is based on transversions only to avoid postmortem damage driving the differences between ancient and modern populations. ROH comparison of ASTF002 and modern breeds is shown in c) for the WGS dataset and d) for the SNPCHP dataset.

The good preservation of the sheep remains sequenced in this study allows us to estimate genetic diversity for the prehistoric and historic populations and to compare them to the same estimates in modern breeds. As aDNA damages and other properties of the data complicate calling diploid genotypes, we chose an approach where we count pairwise differences between individuals from the same group at a set of biallelic sites known to be polymorphic (Skoglund et al. 2014). The result should provide an estimate correlated with the expected heterozygosity in each population and as the data is restricted to transversions, the results should not be driven by postmortem deamination damages. The pairwise mismatch results for modern populations are largely similar to what has been estimated from whole genome resequencing data (Lv et al. 2022) with Asiatic mouflon displaying the highest diversity of all sheep populations, consistent with a domestication bottleneck, and more variation between particular breeds than between continental groups (Fig. 5a). Both ASTF and AKAS show higher levels of diversity than a number of modern European breeds. This signal is even more pronounced when comparing to the set of breeds in the SNPCHP dataset (Fig. 5b) where the ancient groups are significantly higher than all estimates for modern NEST breeds. The difference between the datasets can be explained by the choice of breeds with Gotland sheep (lowest diversity among NEST breeds in WGS) representing the only NEST breed in both datasets. The other NEST breeds in the WGS data are rather large breeds compared to the local NEST breeds in the SNPCHP dataset which all have breeding sizes below 10,000 individuals (Dýrmundsson and Niżnikowski 2010). These results suggest a very recent loss of genetic diversity in many Northern European sheep in the last 500 years.

The relatively high coverage data (11.6X) for ASTF002 also allowed us to call diploid genotypes (Prüfer 2018) and to estimate runs of homozygosity for this individual and compare the results to modern sheep breeds. The general pattern of runs-of-homozygosity (ROH) is comparable to the results for the pairwise mismatches with ASTF002 falling toward the lower end of modern NEST groups but we do not want to draw broader conclusions from this single sample (Fig. 5c and d).

### The Wool Phenotype and Tail Length in the Ancient Sheep Samples

To investigate the fiber quality in the ancient sheep, we checked two loci that were associated with wool fiber thickness or quality in previous studies (Demars et al. 2017; Lv et al. 2022). First, we genotyped the insertion of an antisense *EIF2S2* retrogene into the 3′ untranslated region (UTR) of the gene *IRF2BP2* which likely affects coatedness (Demars et al. 2017). We mapped the sequencing reads of all individuals to modified versions of the Oar3.1 reference genome with and without the insertion (Demars et al. 2017; Rossi et al. 2021). As multiple copies of the retrogene exist in the genome, we can only use reads spanning the insertion breakpoints as diagnostic. Using the reference genome including the insertion, only a single read for all five individuals (from ASTF002) maps across an insertion breakpoint, by only 3 bp out of which two are mismatches to the reference sequence (ASTF001 and ASTF002, supplementary fig. S11, Supplementary Material online). In the modified reference without the insertion, two reads are crossing the breakpoint (supplementary fig. S11, Supplementary Material online). While the number of reads is small in both cases, we do not consider this strong evidence for the presence of the derived allele. The ancestral state was also found at the SNP rs161553028 (supplementary table S8, Supplementary Material online), also located in the 3′ UTR of *IRF2BP2* and the derived allele correlated with thinner fiber according to Lv et al. (2022). Finding the ancestral state for both mutations is not surprising as most investigated modern NEST individuals are also ancestral at these mutations (Lv et al. 2022). While these results suggest some coarser wool in the ancient sheep, we cannot make a statement whether they were used as a source of fiber in addition to meat and potentially milk.

Furthermore, we investigated an insertion in the 5′ UTR of *HOXB13* which is seemingly causal for tail length (Lagler et al. 2022). The reads of our ancient individuals were mapped to a reference assembly without the insertion (Davenport et al. 2022), i.e. the ancestral allele associated with short tails. For all but the lowest coverage AKAS001, we find reads overlapping the insertion breakpoint (supplementary fig. S12, Supplementary Material online), suggesting that they were of the ancestral, short-tail phenotype, similar to modern NEST breeds.

## Discussion

Despite only representing five individuals spread across almost four millennia, the insights gained from our aDNA data highlight the power of studying the recombining part of the genome in temporal data for our understanding of demographic processes. The individuals from the two Baltic islands of Åland and Gotland show remarkable genetic similarities to each other suggesting that they were part of the same gene pool that has been present in the Baltic Sea region for the last four millennia. A long continuity of sheep in the Baltic Sea region since at least the Late Bronze Age has already been suggested based on uniparental data (Niemi et al. 2013; Rannamäe et al. 2016b). Our study now extends this observation by another millennium on the autosomal level.

We see the highest level of similarity between our ancient sheep, including the Late Neolithic, and the modern Åland breed. While the ancient genomes display a high similarity to NEST breeds in general, none of the modern breeds is a direct match for a population with uninterrupted continuity. This suggests that other genetic processes and input from outside of Scandinavia as well as potentially unsampled Northern European populations were involved in the development of these separate breeds during the last few centuries. In the 16th century, these sheep were just old traditional types of sheep in Scandinavia, likely without strong active breeding and selection for specific traits. Now most sheep used for production are crosses between fine wool and heavier meat sheep, and some level of cross-breeding with other breeds has been documented for almost all NEST breeds (Dýrmundsson and Niżnikowski 2010). The drop in genetic diversity after the AKAS individuals lived, i.e. since the 16th to 17th century, can probably be attributed to the development of separate breeds since the 18th century (Wood 1973) which involved more controlled mating among individuals and/or to the reduction in the number of local sheep landraces during the last centuries when larger meat-type breeds became more common. Simultaneously, it is remarkable that the diversity stayed constant between ∼4,000 cal BP and the 16th century CE suggesting that the mating patterns have not changed substantially during this time period. A recent drop in genetic diversity of Northern European sheep has also been found in Bayesian Skyline plots based on uniparental markers although these suggested a lower level of diversity before the Medieval period (Niemi et al. 2018).

The Late Neolithic sheep postdate the arrival of farming practices and sheep in Scandinavia by more than 1,000 years, which would represent the Funnel Beaker culture. The Late Neolithic sheep also postdate the Battle Axe Culture, a Scandinavian variant of the Corded Ware horizon which has been shown to introduce a different type of genetic ancestry from the Pontic–Caspian steppe into human populations (Malmström et al. 2019). The Late Neolithic and Early Bronze Age were turbulent periods showing large transformations in the human gene pool all over Europe (Allentoft et al. 2015; Haak et al. 2015). Similar processes may have taken place in human-dependent species around this time. In horses, the expansion of the so-called DOM2-lineage dates to this time (Fages et al. 2019; Librado et al. 2021) and it is possible that sheep populations have also seen secondary expansions, potentially originating in the Eurasian Steppe and associated with the secondary products revolution and the expansion of wool economies around this time (Sherratt 1983; Chessa et al. 2009; Marciniak 2011). Our current dataset, however, does not allow us to directly test this hypothesis as we lack both Early or Middle Neolithic Scandinavian sheep genomes as well as a description of a potential source gene pool for this secondary expansion. Future studies combining genomic and other bioarcheological data across time and space will offer insights into these questions and processes for sheep in Scandinavia and elsewhere as well as for other domestic species.

## Materials and Methods

### Site Description and Archeological Samples

Stora Karlsö is a small island with several caves located ∼7 km west of the island of Gotland, Sweden. The biggest cave on Stora Karlsö is called Stora Förvar and shows some of the earliest signs of human habitation on Gotland. The cave site Stora Förvar was excavated between 1888 and 1893 (Schnittger and Rydh 1940). Around 9,300 years ago (cal BP), hunter–gatherers, mainly subsisting on marine hunting and fishing, lived in the cave (Lindqvist and Possnert 1997, 1999). The cave was in repeated use except for a hiatus between 7,600 cal BP and 6,000 cal BP when the cave appears to have been abandoned. After ∼6,000 cal BP the cave again was used and some of the oldest dates of domesticated animals have been recorded from the cave around 6,000 cal BP (Lindqvist and Possnert 1997; Rundkvist et al. 2004). After the Neolithic period the cave exhibits a continuous use, but with varying intensity, until the Iron Age and historical times (Schnittger and Rydh 1940). The Stora Förvar cave is ∼25 m deep and at the time of the excavations, the cave was filled with “cultural layers” with a thickness of up to 4 m (Schnittger and Rydh 1940). The cave was excavated in sections (Sv. Parceller, labeled A–I, from the entrance and to the inner part of the cave) and in 0.3 m spits, i.e. the equivalent of a Swedish “foot” (Schnittger and Rydh 1940). Three right radii of sheep from Stora Förvar were included in the study (supplementary table S6, Supplementary Material online). The samples were recovered in section I which was in the innermost parts of the cave where seven layers were noted. Thus, the thickness of the sediments was ∼2.1 m in section I. The section was the last one to be excavated in 1893. The samples originate from layers 4 and 5 which were the deepest layers exhibiting bones from domesticated animals. Layers 6 to 7 in section I exhibit Mesolithic finds but with some recent intrusions of finds, e.g. pottery sherds. Two radiocarbon dates in section I fall in the Mesolithic (Apel et al. 2018). One sample was recovered from the cave wall c. 53 cm above the (excavated) cave floor, i.e. approximately in layer 6. Two sheep bones in section I, also recovered in the cave wall (c. 1.7 and 2 m from the floor) were previously radiocarbon dated to the Early Iron Age, roughly to the second century AD (Apel et al. 2018). So far, the oldest dating of sheep at Stora Förvar is ∼5600 to 6000 cal BP (Rundkvist et al. 2004).

Kastelholm castle is located on the main island of the Åland archipelago, a self-governing Swedish-speaking county of Finland. The castle was built at the end of the 14th century. The oldest written record is from 1,388 and the castle became the administrative center for the Åland Islands. The castle burnt down several times in both the 16th and 17th centuries but was rebuilt and expanded. The landscape was modified and especially the area at the shore closest to the castle. The castle lost its administrative functions in the 1630s and by 1,747 the castle had been abandoned and became a ruin (Palamarz 2004; Kivikero 2020). Over the years many noblemen and their bailiffs held the castle, both Swedish and Danish. Sheep meat, wool, and other products were collected as tax from the archipelago and the castle also had a landed estate nearby with its own husbandry, farming, and fishing (Kivikero 2020). There is also a great deal of correspondence from the noblemen to what is now mainland Sweden as well as to the nobility and priesthood of the cities Tallinn and Riga, capitals of Estonia and Latvia (Hausen 1934). Possibly sheep were imported to Åland from both sides of the Baltic Sea. The first sheep in the Åland Islands are dated to the Late Neolithic period, c. 3850 to 4400 cal BP (Storå 2000). Two right radii from Kastelholm were included in the study (supplementary table S6, Supplementary Material online). The samples from Kastelholm castle were recovered in archeological excavations in 1986 in an area outside and to the east of the Castle (Nordman 1991). The excavations revealed archeological remains that were linked to four different chronological phases. The sampled sheep bones originate from the oldest layers, i.e. phase 1, which consist of a secondarily deposited landfill along the shore east of the castle. The archeological finds show the date to fall around 1500 to 1550 AD, which is in good agreement with the radiocarbon dating of AKAS001 (Table 1).

### Radiocarbon Dating

Samples were radiocarbon dated by the Tandem Laboratory at Uppsala University. Bone samples were mechanically cleaned by scraping and then ground in a mortar. 0.25 M HCl was added and incubated at ambient temperature for 48 h. 0.01 M HCl was added to the insoluble fraction and incubated at 50 °C for 16 h. The soluble fraction was added to a 30 kDa ultrafilter and centrifuged. The retentate was lyophilized. Prior to accelerator determination the Fraction to be dated was combusted to CO_2_ using a Fe-catalyst. Acquired dates were calibrated using atmospheric data from IntCal20 (Reimer et al. 2020) in IOSACAL v.0.4.1 (Costa and Gutiérrez-Roig 2018).

### Sample Preparation and Sequencing

DNA was extracted from samples in a dedicated clean lab by the SciLifeLab aDNA unit at Uppsala University. Before sampling each bone was UV-irradiated (6 J/cm^2^ at 254 nm) on each side. The outer surface was then removed using a Dremel drill. Bones were wiped with first 0.5% bleach and then Milli-Q water using sterile cotton swabs. A small piece of 50 to 100 mg was cut from each bone using a Dremel drill.

DNA was extracted using a modification to the protocol by Yang et al. (1998), where 1 M urea replaced SDS. Extraction blanks were included as negative control. Samples were pretreated with 1 ml of 0.5 M EDTA for 30 min at 37 °C. EDTA was removed, and samples digested with 1 ml of extraction buffer, under rotation at 37 °C for 22.5 to 23 h, and finally at 55 °C for 3.5 to 4 h. The supernatant was collected and concentrated using an Amicon Ultra-4 filter unit (Millipore). The extract was purified using a MinElute PCR purification kit (QIAGEN) according to standard instructions and finally eluted in 110 μl of EB buffer.

Libraries were prepared by the SciLifeLab aDNA unit at Uppsala University. Double-stranded blunt end libraries were prepared according to Meyer and Kircher (2010) with a MinElute PCR purification kit instead of SPRI beads. Libraries were quantified using qPCR in 25 μl reactions with Maxima SYBR green master mix (Thermo Fisher Scientific), 200 nM primer IS7, and 200 nM primer IS8 to determine the number of indexing cycles. Indexing was done in duplicates of 50 μl reactions using 6 μl of DNA library, 5 units of AmpliTaq Gold DNA polymerase (Thermo Fisher Scientific), 1′ GeneAmp Gold buffer (Thermo Fisher Scientific), 2.5 nM MgCl_2_, 250 μM of each dNTP, 200 nM of primer IS4, and 200 nM of indexing primer. PCR was performed as follows: 94 °C for 10 min, 14 to 17 cycles of (94 °C for 30 s, 60 °C for 30 s, 72 °C for 45 s), and 72 °C for 10 min. Duplicates were pooled and purified with AMPure XP beads (Beckman Coulter). Quality of libraries was assessed using an Agilent 2200 Tapestation and Qubit dsDNA HS assay kits (Invitrogen).

Sequencing was done at the SciLifeLab SNP&SEQ Technology Platform at Uppsala University. Libraries were sequenced on an Illumina NovaSeq S4 flow cell using v1 chemistry and 100 bp paired-end reads.

### Data Preprocessing and Mapping

Adapters were removed using CutAdapt v.2.3 (Martin 2011) using the following settings: --quality-base 33 --nextseq-trim = 15 --overlap 3 -e 0.2 --trim-n --minimum-length 15:15. Reads were merged using FLASH v1.2.11 (Magoč and Salzberg 2011) with the following settings: --min-overlap 11 --max-overlap 150 --allow-outies. The quality of the reads was assessed using FastQC v0.11.9 (Andrews 2010). To corroborate that the sequences belonged to sheep, FastQScreen v.0.14.0 (Wingett and Andrews 2018) was run with a set of reference libraries composed of common contaminants plus those of a goat and a sheep.

Merged reads were mapped to the Texel sheep reference 3.1 (Oar3.1) using Bowtie2 v.2.3.5 (Langmead and Salzberg 2012) using the local alignment option, a mismatch penalty (--mp) of 4 and allowing for 1 mismatch in the seed alignment (-N 1). Other parameters were set as default. BAM files were sorted using SAMtools v.1.14 (Danecek et al. 2021) view and sort, respectively, using the standard settings. PCR duplicates were removed using DeDup v.0.12.8 (Peltzer et al. 2016). The merged reads were also mapped to the Texel sheep reference genome 4.0 (Oar4.0; Consortium et al. 2010) containing the mitochondrial sequence from NCBI accession number NC_001941.1 using BWA aln v.0.7.17-r1188 (Li and Durbin 2009) and the nondefault settings: -l 1024 -n 0.01 -o 2. Mapped reads were filtered using a minimum mapping quality of 30 and a minimum fragment length of 30 bp. These reads were sorted using SAMtools sort with standard settings. Duplicate reads were removed using DeDup v.0.12.8 (Peltzer et al. 2016) with standard settings. BAM files with duplicates removed were merged per individual using SAMtools merge and indexed using SAMtools index with standard settings for both Oar3.1 and Oar4.0. In order to validate the authenticity of the aDNA data, misincorporation patterns were calculated and visualized using mapDamage v2.0.9 (Jónsson et al. 2013), with standard settings. Overall mapping quality check was performed on both Oar3.1 and Oar4.0 with QualiMap v2.2.1 (Okonechnikov et al. 2016), and all quality control analyses were visualized using MultiQC v1.12 (Ewels et al. 2016).

### Molecular Sex Determination and Mitochondrial Haplotyping

Coverage was calculated using QualiMap v2.2.1 (Okonechnikov et al. 2016) on reads mapped to the modified Oar4.0 reference. Sex was determined by comparing the average coverage in the autosomes to the average coverage in the X chromosome using SAMtools depth, where a similar coverage was considered female, and around half the coverage in the X chromosome was considered male.

Mapping Iterative Assembler (MIA) v.5a7fb5a (Stenzel 2013) was used to call consensus sequences for mitochondria in ancient samples. To remove reference bias, the Mouflon mitochondrial reference was used as well as a substitution matrix specific for aDNA. Consensus fasta-sequences were then assembled using sites with coverages higher than 3×, 15×, and 50× as cutoff values, using Map Assember in MIA. As AKAS-001 has low coverage, a minimum 2× consensus was instead assembled for that sample.

As a measure of quality control, all mitogenome fasta files were aligned using MAFFT v.7.407 (Katoh and Standley 2013) with the standard settings, and inspected visually. For AKAS-001 the minimum 2× consensus fasta was chosen to call haplotypes, for AKAS-002 15× and for all ASTF samples the 50×. Haplotypes were assigned using MitoToolPy-Seq from MitoTool v.943ce25 (Peng et al. 2015) with settings: -s sheep -r whole.

### Reference Data and Genotyping

To obtain an unbiased genomic reference panel, all known SNP from nine individuals of six different wild sheep species were collected from dbSNP (supplementary table S1, Supplementary Material online). These SNPs were filtered to remove all multiallelic and duplicated SNPs and using the following settings in PLINK: --mind 0.1 --maf 0.05 --geno 0.1 --hwe 0.01. One hundred and eighty-eight published modern sheep genomes were selected from publications (Naval-Sanchez et al. 2018; Deng et al. 2020; Li et al. 2020) to cover a wide range of modern breeds (supplementary table S2, Supplementary Material online). Raw sequencing reads for these individuals were downloaded and mapped with bwa mem (Li 2013). PCR duplicates were marked with Picard (http://broadinstitute.github.io/picard). SNPs were called and merged in these 188 sheep using GATK HaplotypeCaller (Van der Auwera and O’Connor 2020) using settings: --genotyping-mode GENOTYPE_GIVEN_ALLELES --output-mode EMIT_ALL_SITES. This set of SNPS was filtered using GATK VariantFiltration (Van der Auwera and O’Connor 2020) with the following filters: QD < 2.0, FS > 60.0, MQ < 40.0, SOR > 3.0, QUAL < 30.0, MQRankSum < −12.5, ReadPosRankSum < −8.0. This resulted in a reference panel of 2785430 SNPs. This reference panel was subset to include only Eurasian sheep breeds and Iranian Mouflons, and then filtered to remove low-quality SNPs, using PLINK v.1.90b4.9 (Purcell et al. 2007; Chang et al. 2015) with settings: --geno 0.01 --maf 0.01 --chr-set 26 no-y no-xy --allow-no-sex. Which left 161 modern individuals and 2100214 SNPs for analysis. We call this dataset WGS throughout the study.

To call SNPs in the ancient samples from sequences aligned to Oar 4.0, we used SAMtools mpileup v.1.14 (Danecek et al. 2021) with base alignment quality turned off and nondefault settings: -B -q 30 -Q 30. At each position, a random read was drawn to code the individual as homozygous for that allele, at that position. A/G and C/T SNPs were coded as missing in all individuals to minimize the effect of cytosine deamination.

To further situate the ancient samples in the Baltic Sea area a dataset consisting of European breeds genotyped on the Illumina Ovine Infinium HD 600K chip was prepared (supplementary table S3, Supplementary Material online). Data from Rochus et al. (2018) was downloaded from Zenodo (Moreno-Romieux et al. 2017). Data from Rochus et al. (2020a) was downloaded from DRYAD (Rochus et al. 2020). Åland sheep data from Cao et al. (2021) was acquired from the authors. Data for 16 Russian sheep breeds from Igoshin et al. (2022) was downloaded from Figshare (Larkin and Zinovieva 2021). The reference datasets were merged and filtered with PLINK. First, duplicated positions and individuals were removed, and all heterozygous haploid variants were set to missing. Second, the dataset was filtered to remove low-frequency SNPs with settings: --geno 0.01 --maf 0.01 --allow-extra-chr --allow-no-sex. This left 1,010 individuals and 484,428 SNPs for analysis. SNPs for the ancient samples were again called as described above with the difference that reads aligned to Oar 3.1 were used. We call this dataset SNPCHP throughout the study.

As an outgroup, we also added pseudohaploid genotype calls from NGS data for 19 Iranian Asiatic Mouflon from the NextGen project (Ensembl 2022) which had been downloaded from the European Nucleotide Archive (ENA 2022). The genotype calls were performed the same way as for the ancient samples.

### Exploratory Population Genetic Analysis

To prepare the dataset for PCA and ADMIXTURE data was pruned for SNPs in linkage disequilibrium (LD) to remove SNPs that were highly correlated, using PLINK with settings: --indep-pairwise 200 25 0.4 --chr-set 26 no-y no-xy --allow-no-sex. This left 578,285 SNPs of the WGS dataset and 275,544 SNPs of the SNPCHP dataset for analysis.

We run a PCA to investigate clustering in the data, where ancient samples were projected on a reference panel, using smartpca v.16000 in eigensoft v.7.2.1 (Patterson et al. 2006; Price et al. 2006). To account for ancient individuals being possible outliers, as well as to deal with their amount of missing data these settings were used: altnormstyle: NO, numoutlieriter: 0, killr2: NO, numoutlierevec: 0, lsqproject: YES, shrinkmode: YES.

To further explore clustering with ADMIXTURE (Alexander and Lange 2011), the LD-pruned data set was converted to tped format using PLINK with settings: --recode transpose --chr-set 26 no-y no-xy --allow-no-sex and pseudo-haploidized by randomly choosing one allele at each site per individual in a tped file and coding the individual homozygous for that allele in that position. Pseudo-haploidizing the reference panel individuals reduces sample-specific drift in the ancient individuals that would solely be due to technical issues with the aDNA data. The pseudo-haploidized dataset was converted back to bed format using PLINK with settings: --make-bed --chr-set 26 no-y no-xy --allow-no-sex. ADMIXTURE v.1.3.0 (Alexander et al. 2009) was run, using standard settings and for K levels 2 to 10 with 20 different random seeds for each K.

### *f* Statistics and Diversity

Outgroup *f*_3_ statistics were calculated in ADMIXTOOLS v.2.0.0 (Maier et al. 2023) using the function qp3pop with standard settings and the nonpruned dataset described above without precalculating *f*_2_ statistics. The results were visualized using ggplot2 v.3.3.5 (Villanueva and Chen 2016) in R v.4.0.3 (R Core Team 2020). Conditional nucleotide diversity per group was calculated using POPSTATS (Skoglund et al. 2015), restricting the analysis to transversions (--notransitions) and allowing for more than 23 chromosome pairs (--not23).

Considering that the amount of sequencing data for ASTF-002 is relatively high for an ancient sample (11.6X), we decided to call diploid genotypes for this individual. We first realigned reads around indels using GATK (McKenna et al. 2010). Diploid genotypes were then called with the dedicated aDNA caller snpAD v0.3.4 (Prüfer 2018) restricting to reads with mapping qualities and base qualities of at least 30. Calls were performed separately for the BAM files aligned to Oar 3.1 and 4.0. Only sites with between 5 and 28 reads and genotype quality of at least 30 were considered for the analysis of ROH. The diploid calls were merged with the modern reference data sets using plink excluding triallelic sites. ROH were then estimated using plink and the parameters --homozyg --homozyg-density 50, --homozyg-gap 1000, --homozyg-kb 500, --homozyg-snp 100, --homozyg-window-threshold 0.02, --homozyg-window-snp 100, --homozyg-window-het 1, --homozyg-window-missing 10, --allow-extra-chr, and --chr-set 26.

### Admixture Graphs and Continuity

We constructed admixture graphs from a reduced dataset to further investigate how ancient samples and NEST breeds are related. The SNPCHP data set was subset to include only Mouflons, Swedish NEST breeds, and Russian breeds. Five hundred and two individuals remained for the analysis. The data set was converted to VCF using PLINK and all sites with missing calls were removed with settings: --allow-no-sex --allow-extra-chr --recode vcf-iid --geno 0 and all modern genomes were pseudo-haploidized to match the wild mouflons and the ancient samples. Then the VCF was in turn converted to.tmix format using vcf2treemix.py (Silva 2017). The dataset was then run on OrientAGraph v1.0 (Molloy et al. 2021), with a fixed seed to retain the same starting tree structure for each run, for migration edges 0 to 3 (supplementary fig. S7, Supplementary Material online), chosen as three edges allow for reasonable running time, using settings: -seed 32295 -mlno -allmigs and setting the Asiatic Mouflons as the root of the tree. Trees and residuals were visualized in R v.4.0.3 (R Core Team 2020) using the packages RColorBrewer (Neuwirth 2014) and R.utils (Bengtsson 2021) with the functions plot_tree provided in TreeMix (Pickrell and Pritchard 2012; supplementary fig. S7, Supplementary Material online).

As a complementary approach, we employed qpGraph (Patterson et al. 2012) to reconstruct admixture graphs. We used the find_graphs function in ADMIXTOOLS v.2.0.0 (Maier et al. 2023) to explore the graph space. *f*_2_ statistics were calculated using f2_from_geno allowing for 15% missingness per SNP and more than 22 chromosome pairs. find_graphs was then run with Asiatic mouflon as an outgroup for 1,000 different random seeds and the parameters stop_gen = 10,000, stop_gen2 = 30, and plusminus_generations = 10. We explored between 0 and 5 migration events. Graph fits were compared using the qpgraph_resample_multi and compare_fits functions (supplementary table S7, Supplementary Material online).

In order to test if there has been direct population continuity within the Baltic sheep population through the time period covered by our samples, we used a maximum likelihood ratio test described in Schraiber (2018) (https://github.com/Schraiber/continuity). We used this explicit test for population continuity both on NEST and non-NEST breeds with three or more samples from the WGS and the SNPCHP datasets. In order to polarize the ancestral and derived alleles of our modern datasets, we used a Caphi goat (ENA accession ERR219543) and an Inner Mongolia cashmere goat (SRA accession SRR5557418) mapped to Oar4.0 and Oar3.1, respectively, as the outgroup species. Homozygous alleles in the goat were assigned as ancestral variants. Data was then filtered for high and low coverage sites with the default cutoffs and then two models were fitted to each dataset: one assuming continuity and the other assuming independent populations. The resulting likelihoods were then used to calculate the likelihood ratio statistic.

### Phenotypic Analysis

To investigate if the ancient samples in this study had or lacked the insertion polymorphism causative of a fine wool phenotype, reads were mapped to two modified Oar3.1 references. One containing the full insertion and one lacking the full insertion according to Demars et al. (2017) and Rossi et al. (2021). Merged reads from the samples were mapped with BWA aln v.0.7.17-r1188 (Li and Durbin 2009). These reads were sorted using SAMtools v.1.14 (Danecek et al. 2021) sort with standard settings. Duplicate reads were removed using SAMtools markdup. Samples were then inspected visually in IGV v.2.11.4 (Robinson et al. 2011) to find out how many reads crossed the breakpoints of the insertion and the point in the genome lacking the insertion where the insertion would be situated, according to Rossi et al. (2021). Additionally, the alleles of ancient samples at SNP rs161553028, which is associated with fiber diameter (Lv et al. 2022), were visually inspected in IGV v.2.11.4 (Robinson et al. 2011) in the reads aligned to Oar3.1.

Additionally, we investigated the presence/absence of an insertion in the 5′ UTR of *HOXB13* which is seemingly causal for tail length (Lagler et al. 2022). We followed the approach by Lagler et al. (2022) and mapped all reads to the ARS-UI_Ramb_v2.0 assembly (Davenport et al. 2022) which carries the ancestral allele (no insertion) in contrast to Oar3.1 and Oar4.0. We used the bwa-mem mapping algorithm (Li 2013) as it performs soft-clipping, i.e. reads from an individual carrying the insertion would map to one side of the insertion while the rest of the read, which does not match the reference, would be clipped. As this insertion, in contrast to the antisense *EIF2S2* retrogene into the 3′ UTR of the gene *IRF2BP2*, does not have similarities to other parts of the genome, we can test for its presence or absence by mapping to a single reference assembly.

## Supplementary Material

evae114_Supplementary_Data

## Data Availability

Raw sequence data and aligned reads for the five new ancient individuals are available through the European Nucleotide Archive under accession number PRJEB59481. Genotype calls for the ancient individuals are made available on Zenodo (10.5281/zenodo.10978872).
