# Supplementary material for: Ancient Sheep Genomes Reveal Four Millennia of North European Short-Tailed Sheep in the Baltic Sea Region
Source: Genome Biol Evol. 2024 May 25;16(6):evae114. doi: 10.1093/gbe/evae114 (PMC11162877; doi:10.1093/gbe/evae114)
Supplement: evae114_Supplementary_Data [file evae114_supplementary_data.zip › Supplementary Figures Baltic Sheep.pdf]

Ancient Sheep Genomes reveal four Millennia of North European Short-Tailed Sheep in the Baltic Sea region

Supplementary Figures

Martin NA Larsson[1,†], Pedro Morell Miranda[1,†], Li Pan[1], Kıvılcım Başak Vural[2], Damla Kaptan[2], André Elias Rodrigues Soares[1], Hanna Kivikero[3], Juha Kantanen[4], Mehmet Somel[2], Füsun Özer[5], Anna M Johansson[6], Jan Storå[7], Torsten Günther[1,*]

1 Human Evolution, Department of Organismal Biology, Uppsala University, Sweden
2 Department of Biological Sciences, Middle East Technical University, Ankara, Turkey
3 Department of Culture, University of Helsinki, Helsinki, Finland
4 Natural Resources Institute Finland, Jokioinen, Finland
5 Department of Anthropology, Hacettepe University, Ankara, Turkey
6 Department of Animal Biosciences, Swedish University of Agricultural Sciences, Uppsala, Sweden
7 Osteoarchaeological Research Laboratory, University of Stockholm, Stockholm, Sweden

AKAS-001 out.extendedFrags.fastq.sorted

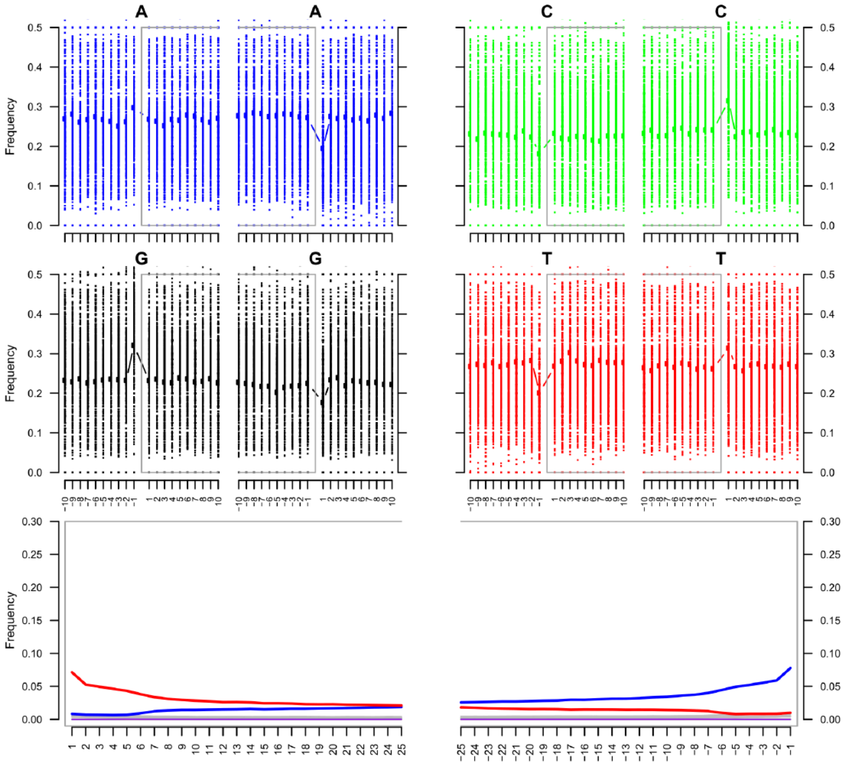

AKAS-002 out.extendedFrags.fastq.sorted

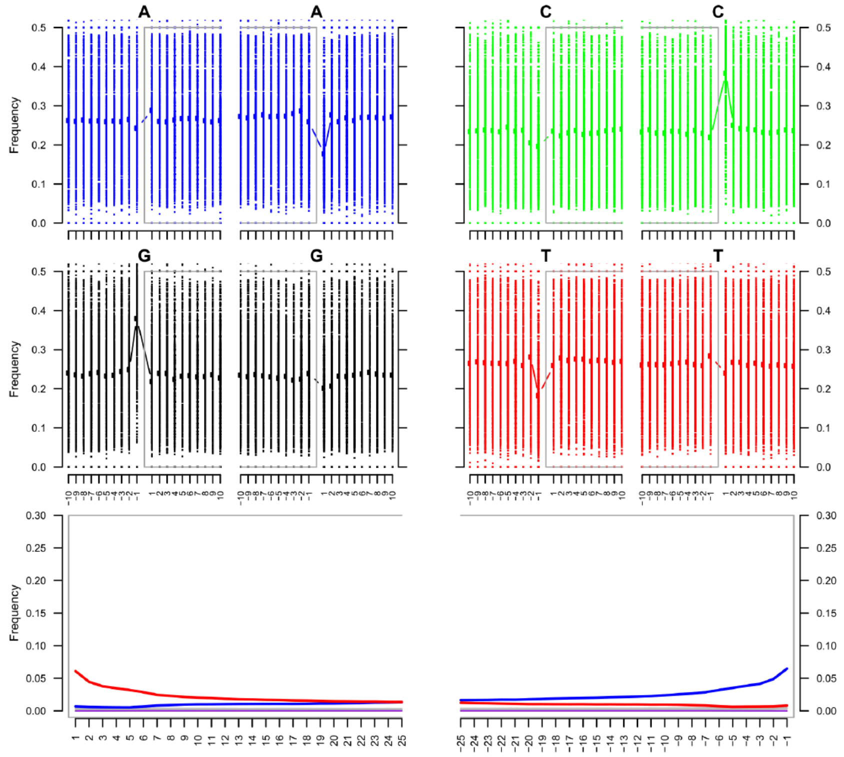

ASTF-001 out.extendedFrags.fastq.sorted

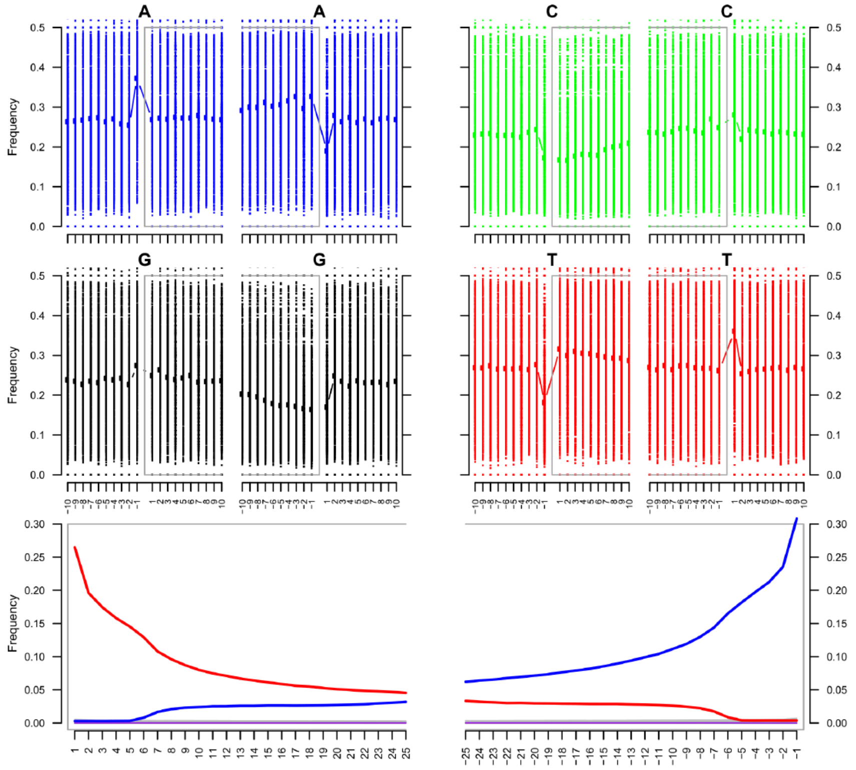

ASTF-002 out.extendedFrags.fastq.sorted

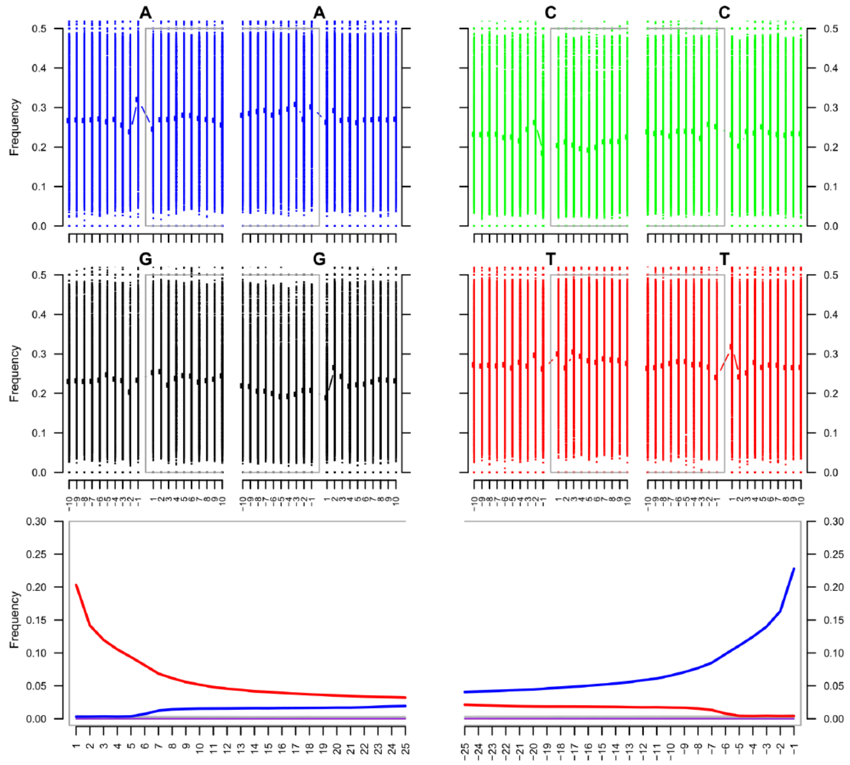

ASTF-003 out.extendedFrags.fastq.sorted

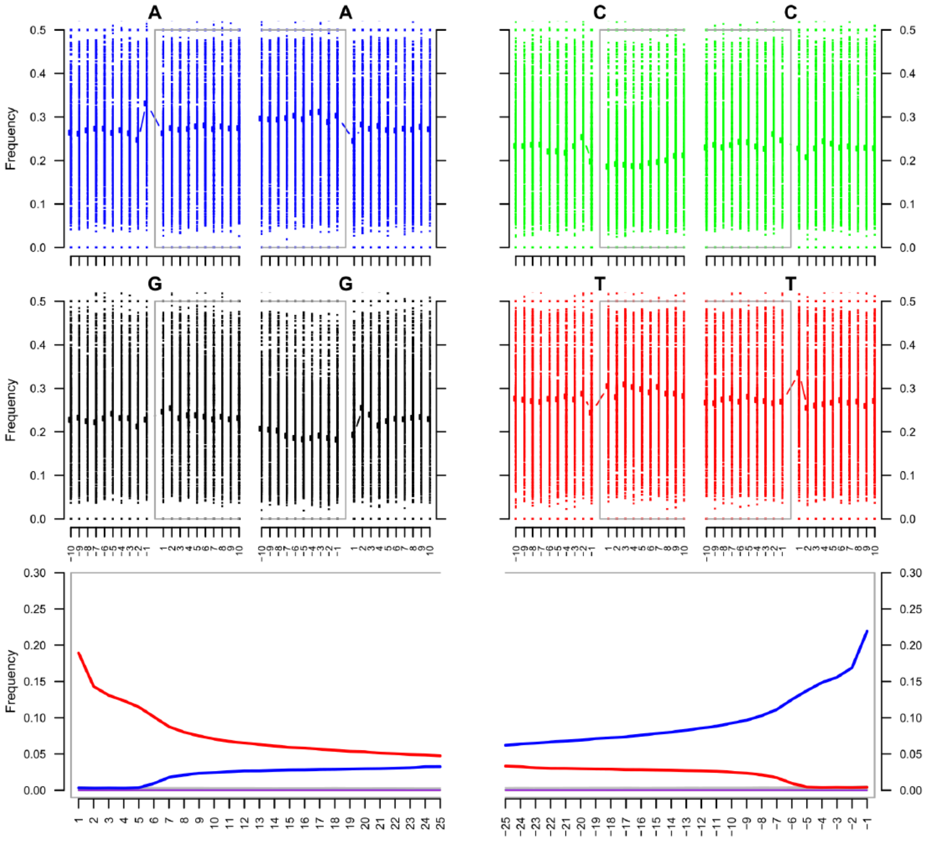

Supplementary Figure 1: mapDamage misincorporation plots for the 5 ancient samples.

ASTF-001

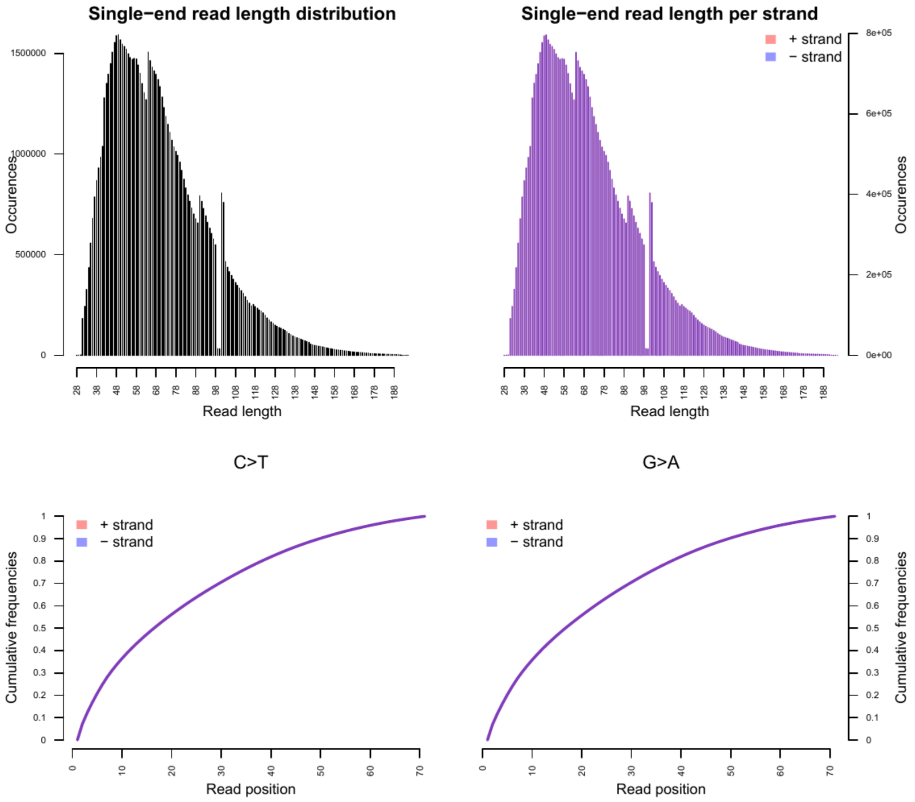

ASTF-002

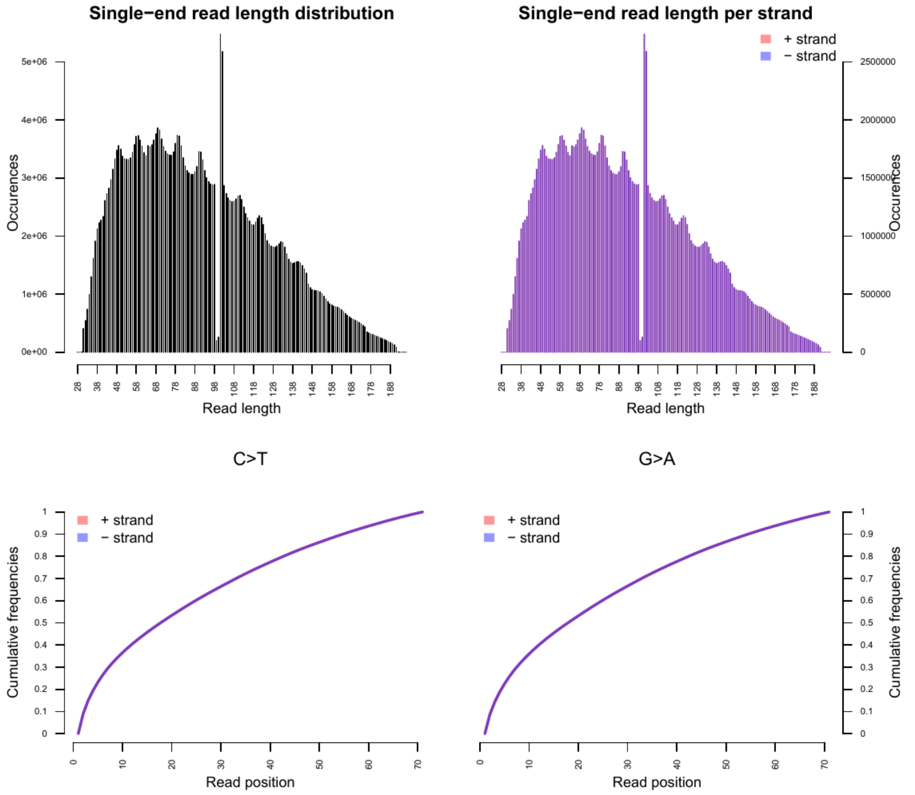

ASTF-003

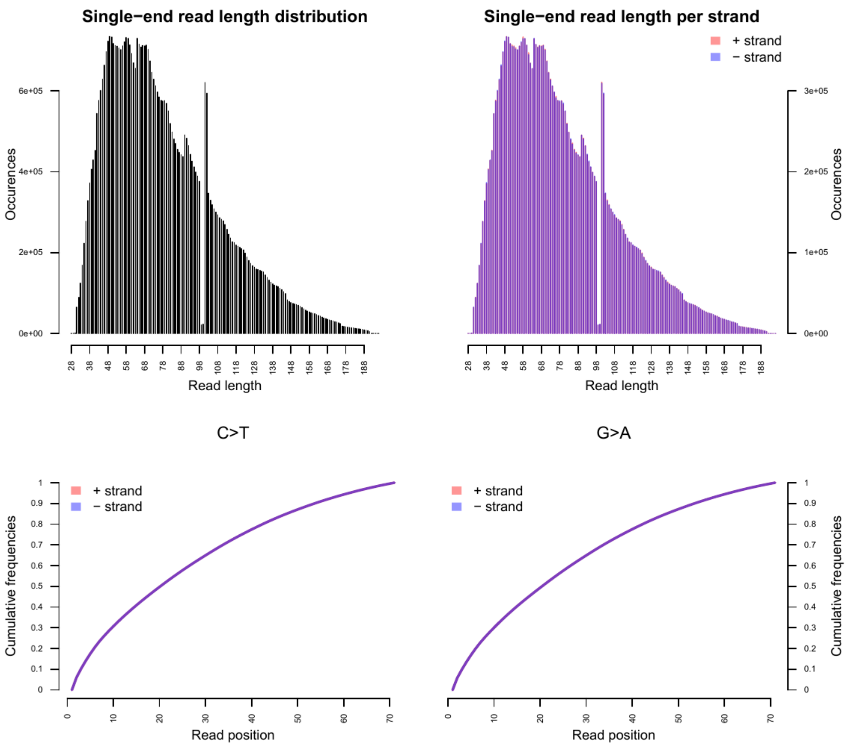

AKAS-001

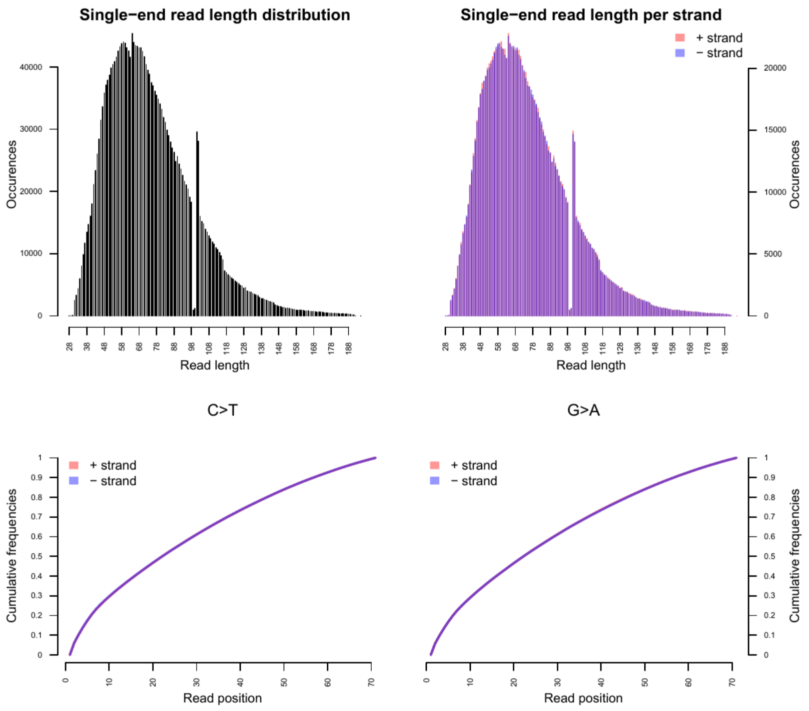

AKAS-002

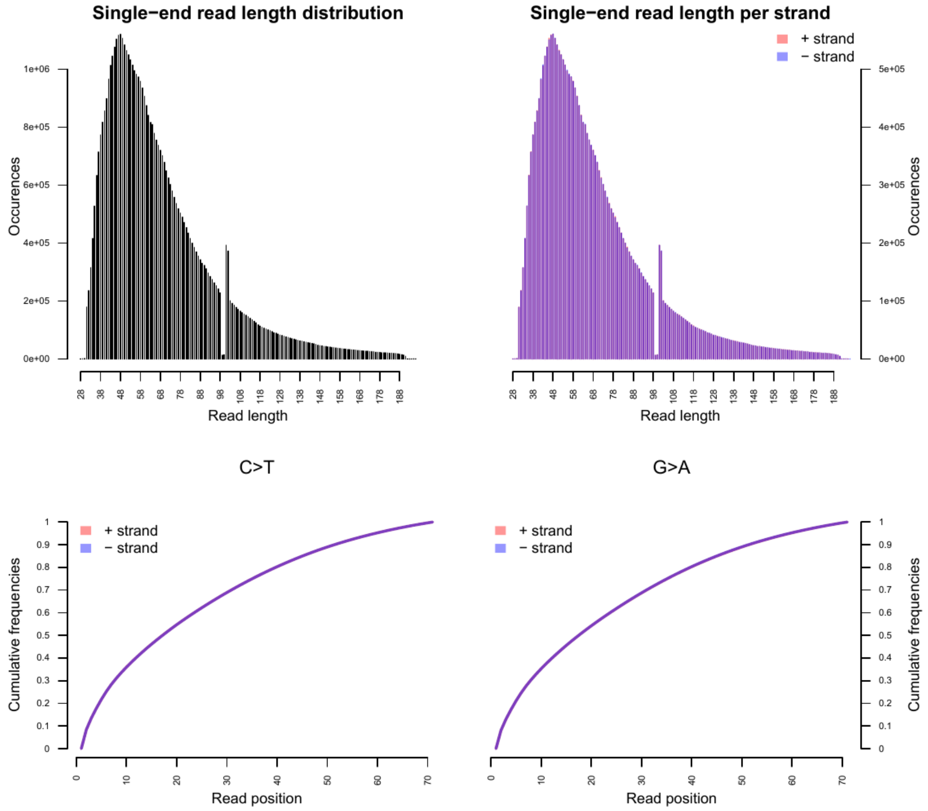

Supplementary Figure 2: mapDamage fragment length plots for the 5 ancient samples.

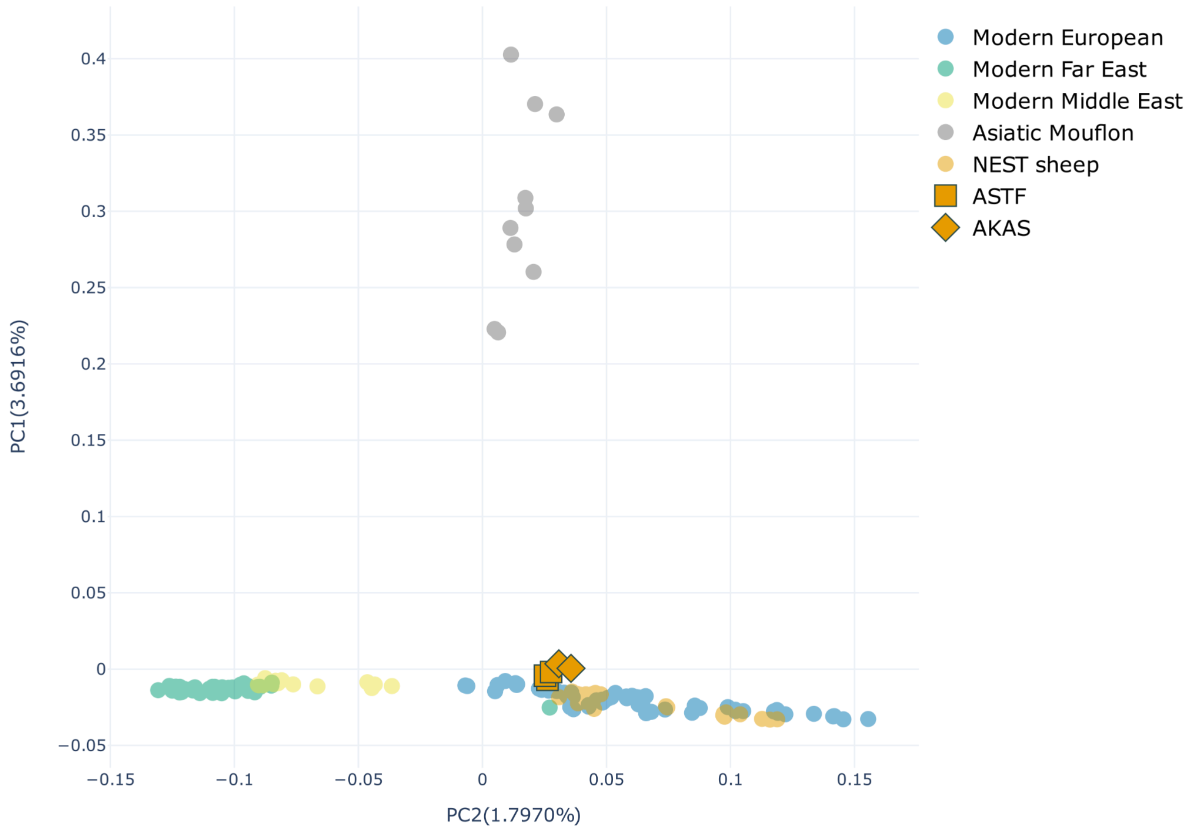

Supplementary Figure 3: WGS PCA showing PC 1 vs PC 2, percentage of explained variation is shown within parentheses of axis titles.

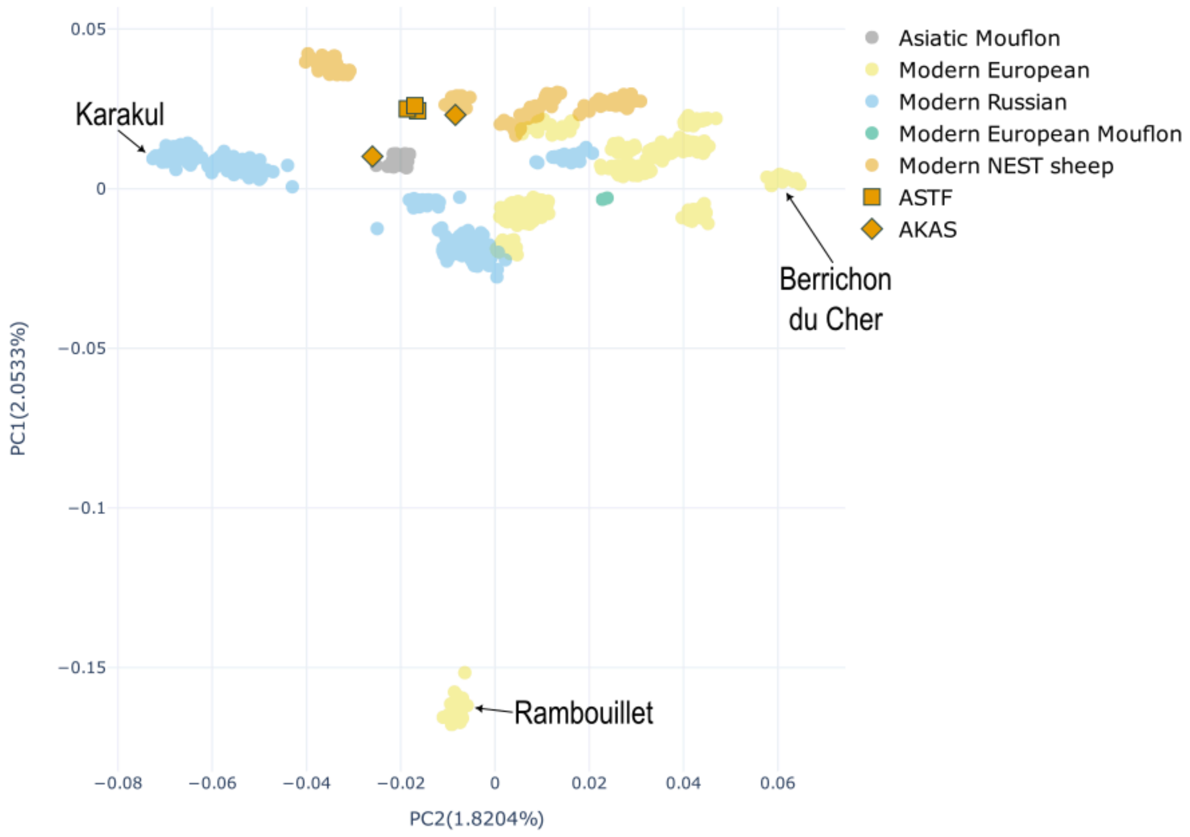

Supplementary Figure 4: SNPCHP PCA showing PC 1 vs PC 2, percentage of explained variation is shown within parentheses of axis titles.

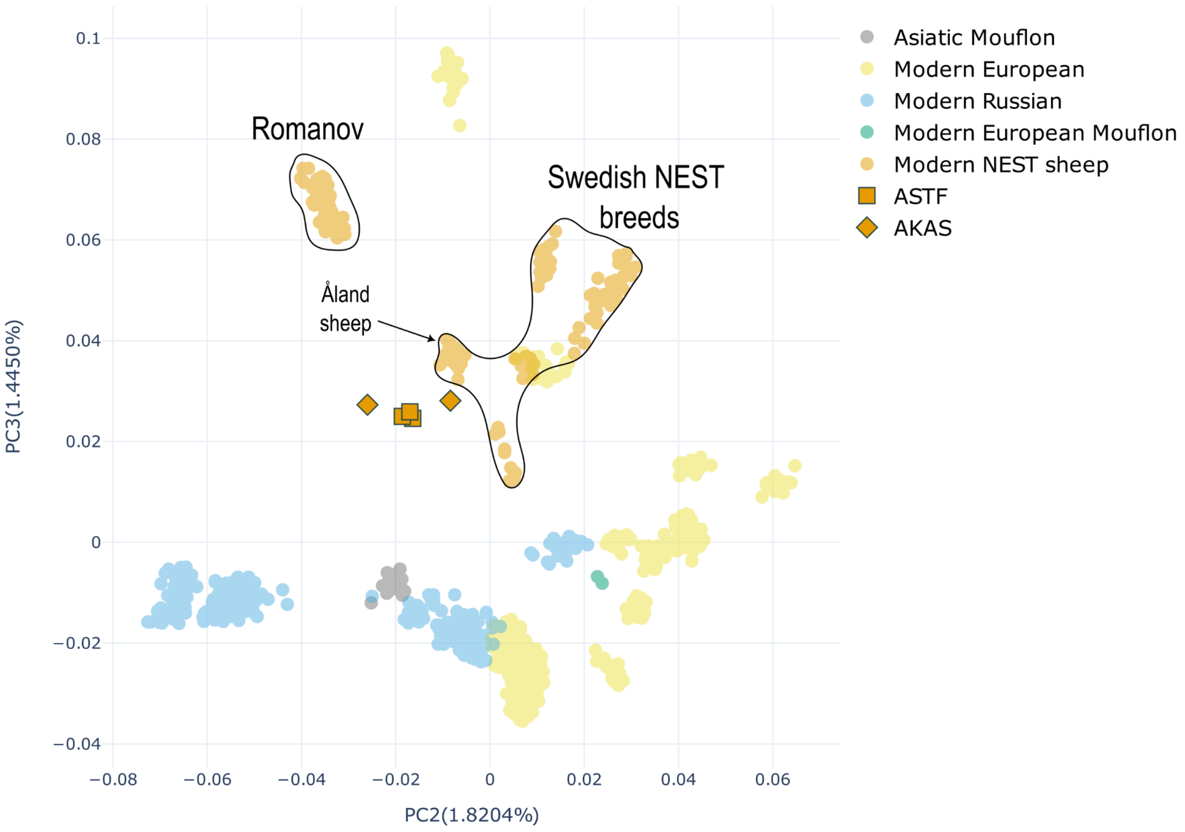

Supplementary Figure 5: SNPCHP PCA showing PC 2 vs PC 3, percentage of explained variation is shown within parentheses of axis titles. The Swedish NEST breeds, Romanov and the ancient samples are pointed out.

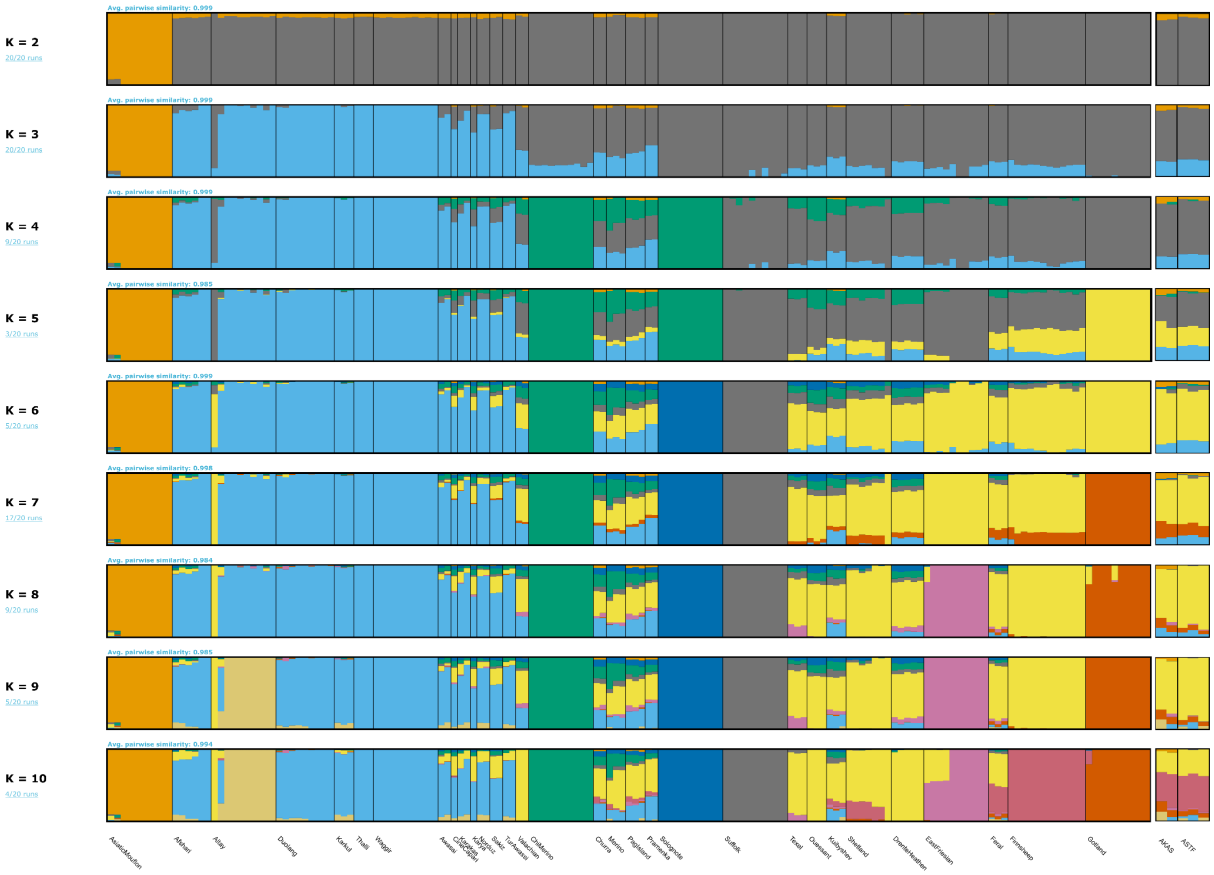

Supplementary Figure 6: WGS Results of 20 ADMIXTURE runs combined by PONG, Vertical bars indicate each individual's genetic variation belonging to a certain cluster, individuals are grouped by breed.

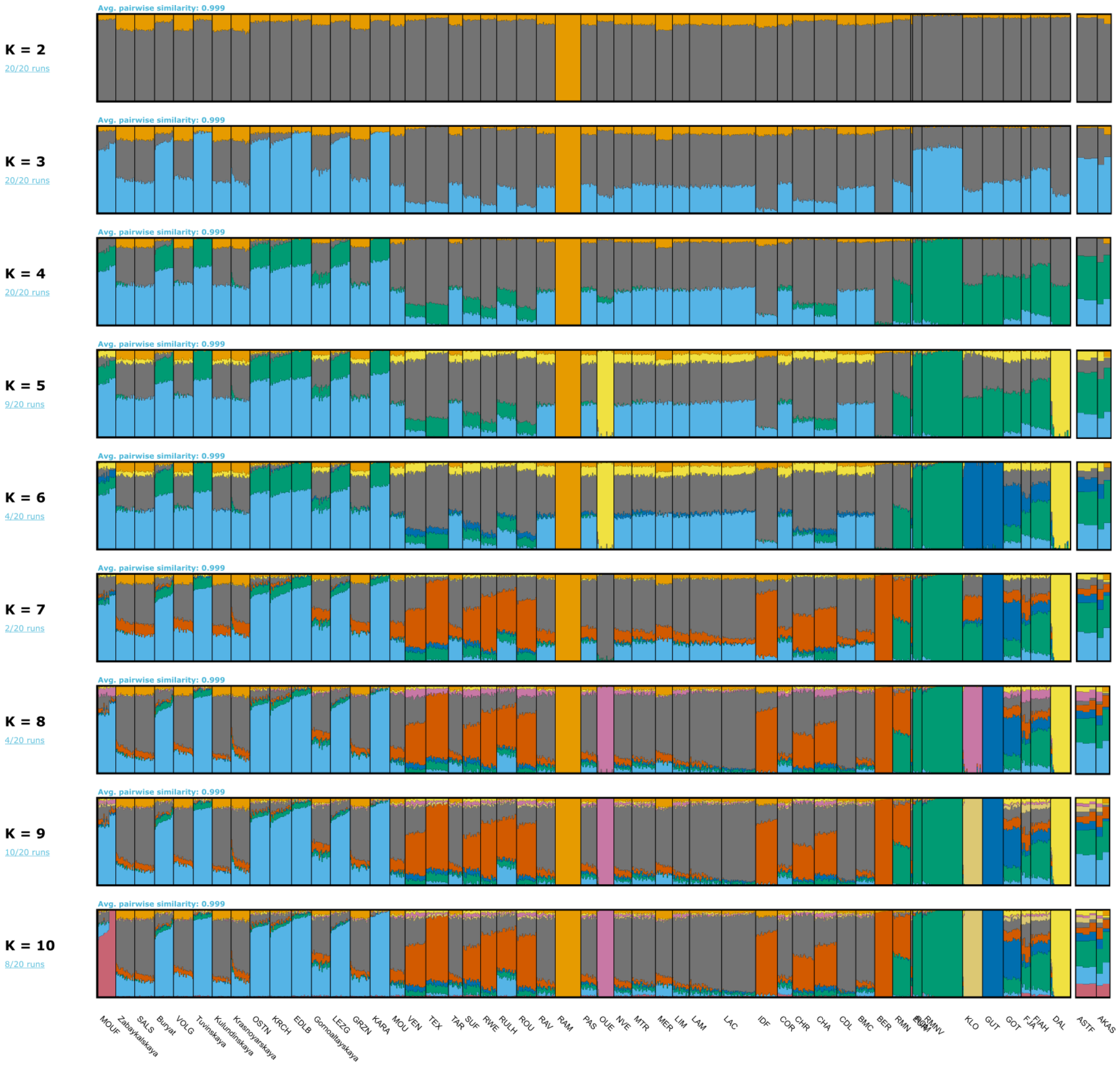

Supplementary Figure 7: SNPCHP Results of 20 ADMIXTURE runs combined by PONG, Vertical bars indicate each individual’s genetic variation belonging to a certain cluster, individuals are grouped by breed.

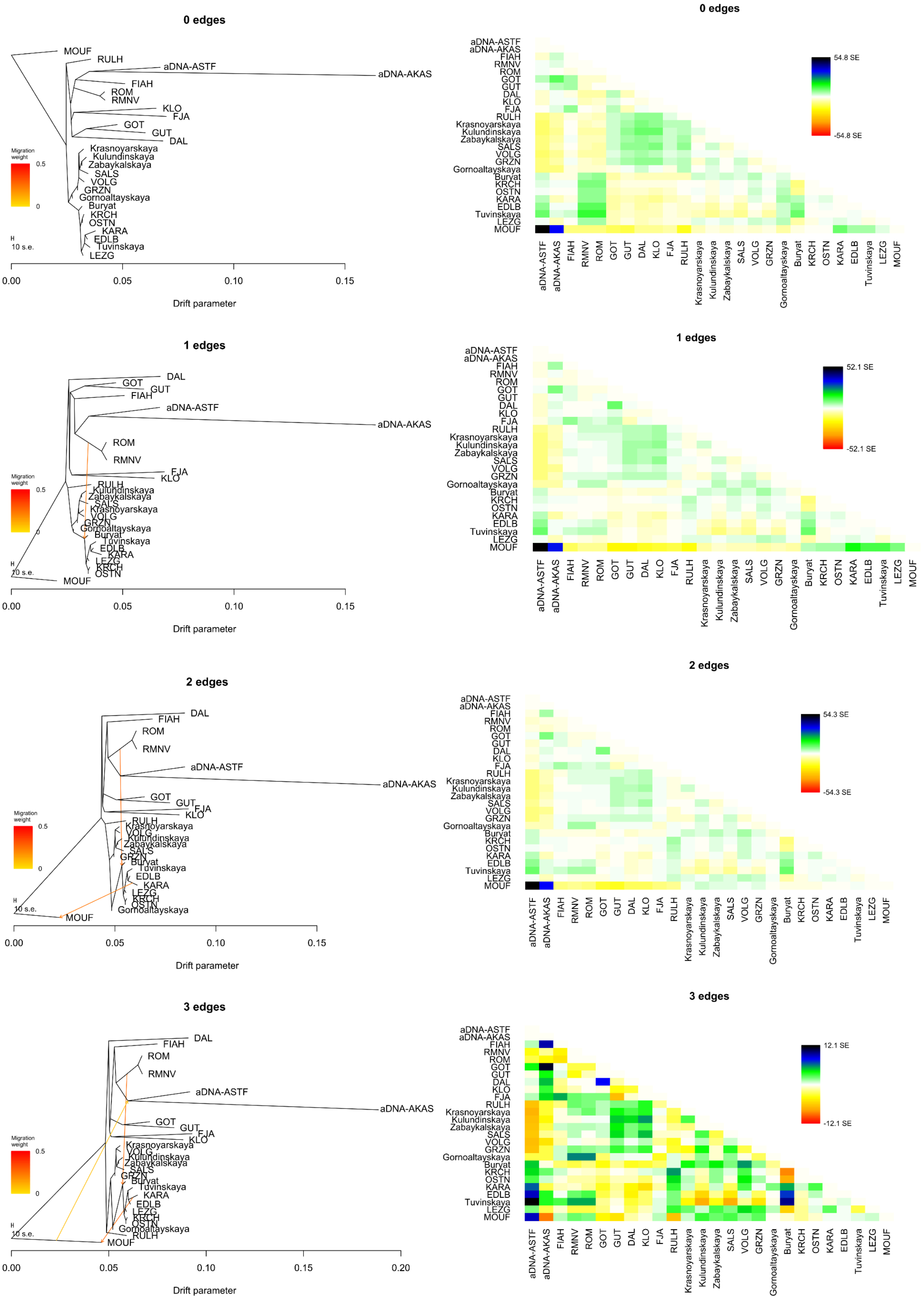

Supplementary Figure 8: OrientAGraph trees from the SNPCHP Panel, 0-3 migration edges, All Russian and Swedish breeds in SNPCHP panel, Asiatic mouflon as well as Ancients.

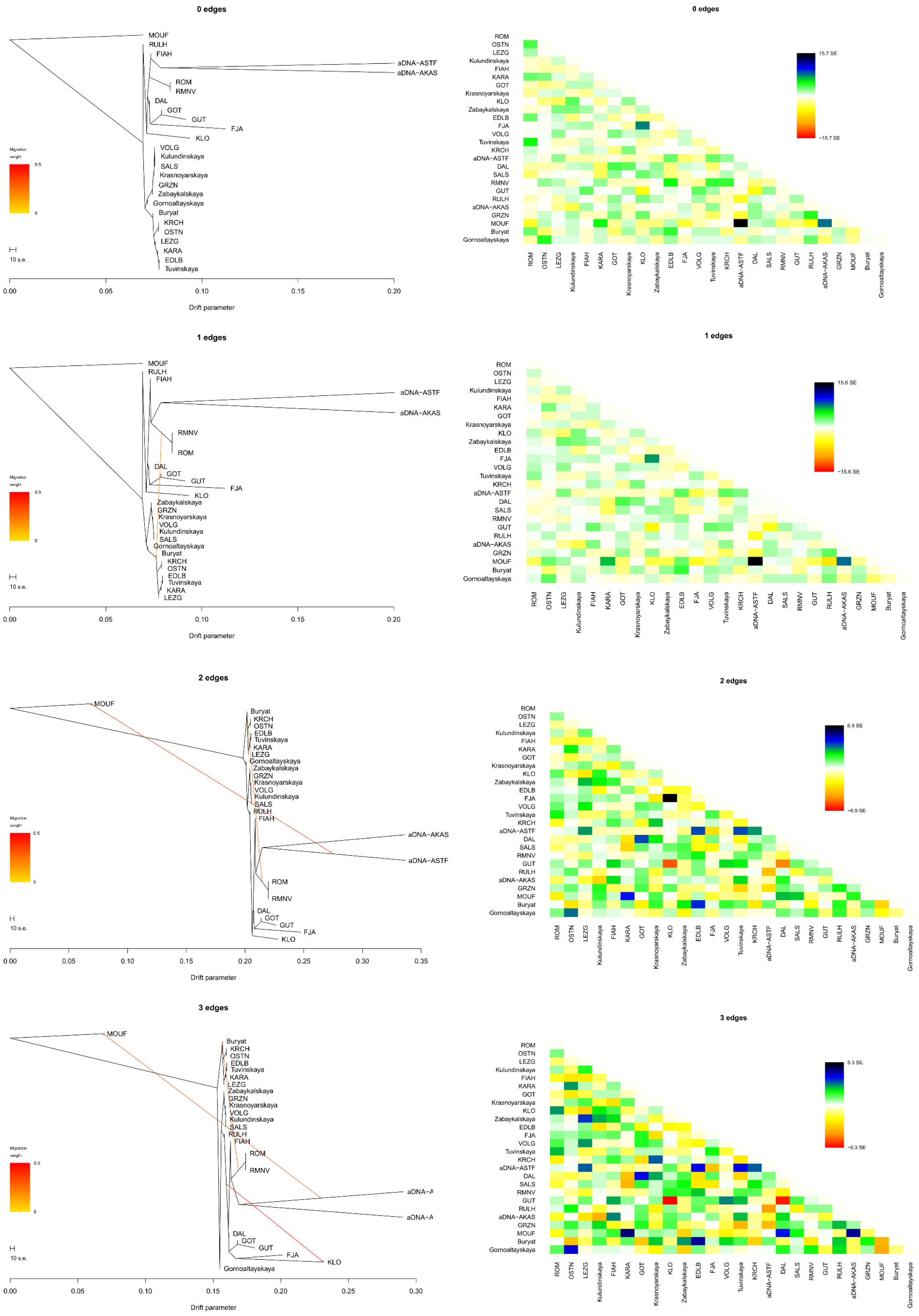

Supplementary Figure 9: OrientAGraph trees from the SNPCHP Panel using only one random individual from each modern population and the highest coverage sample of the ancient sites, allowing for 0 to 3 migration edges.

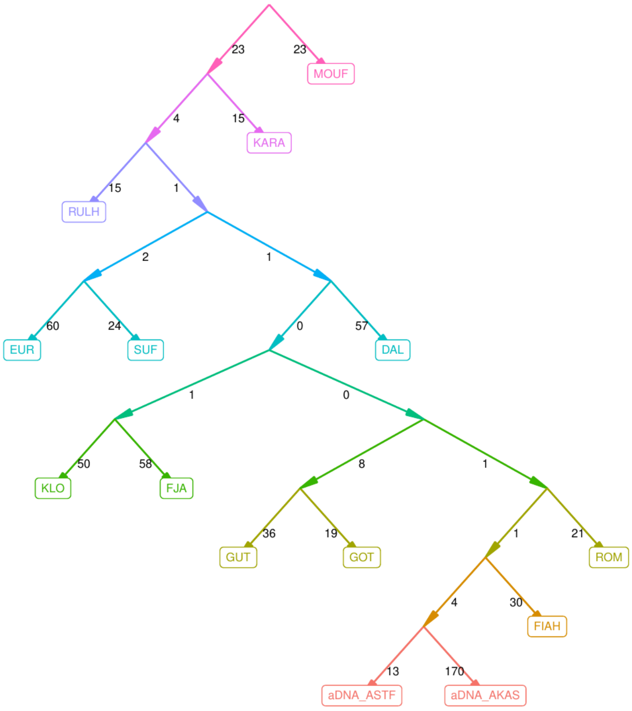

Supplementary Figure 10a: Best fitting admixture graph for m=0 and without constraints.

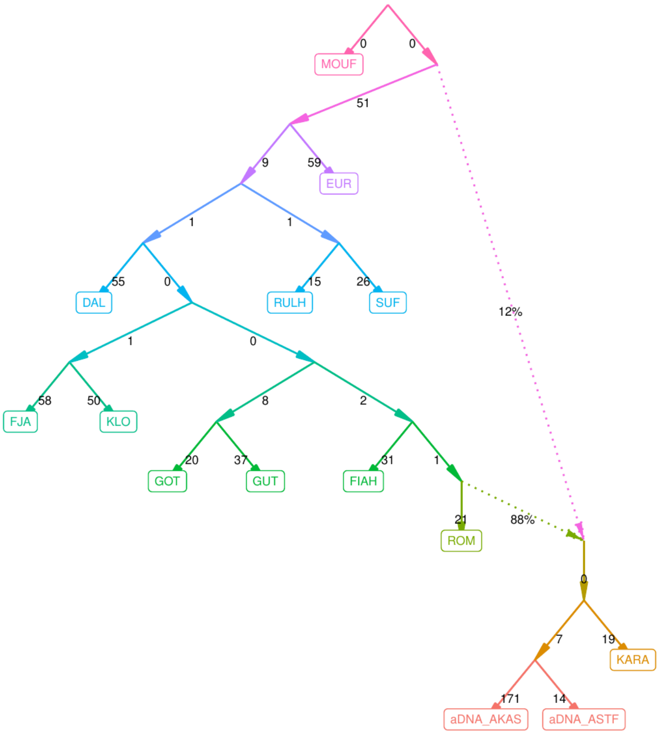

Supplementary Figure 10b: Best fitting admixture graph for m=1 and without constraints.

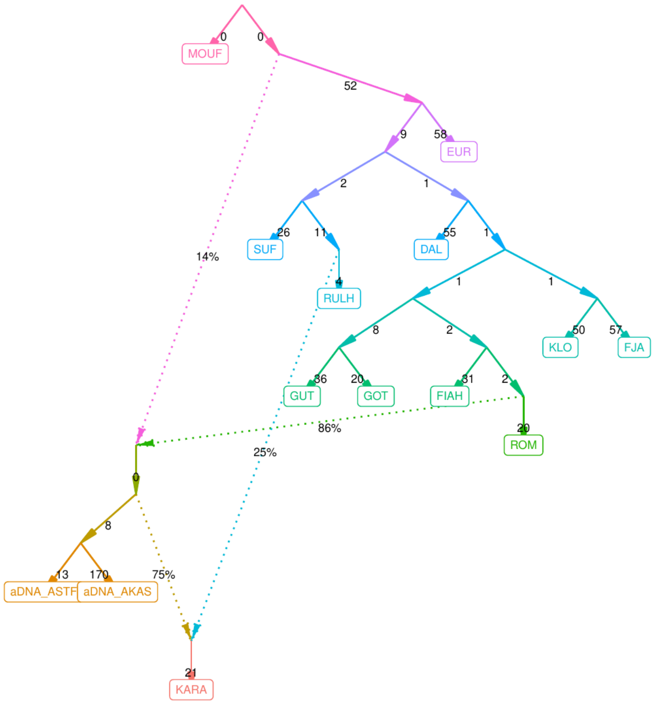

Supplementary Figure 10c: Best fitting admixture graph for m=2 and without constraints.

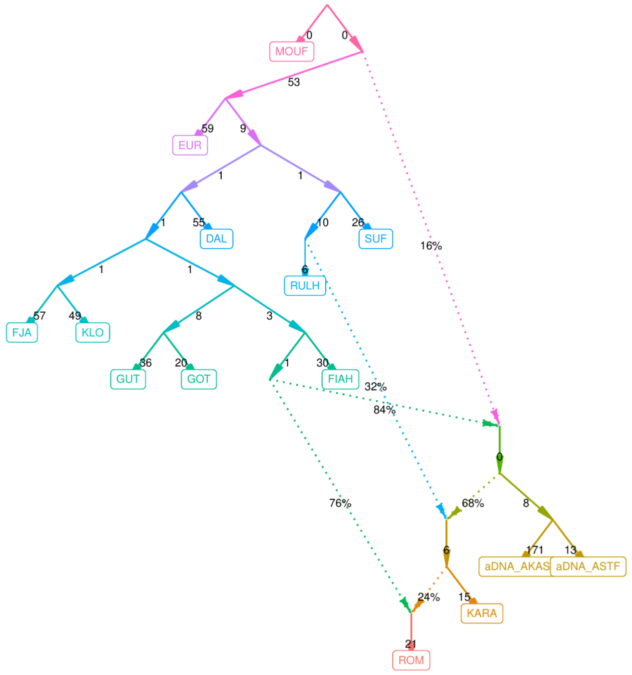

Supplementary Figure 10d: Best fitting admixture graph for m=3 and without constraints.

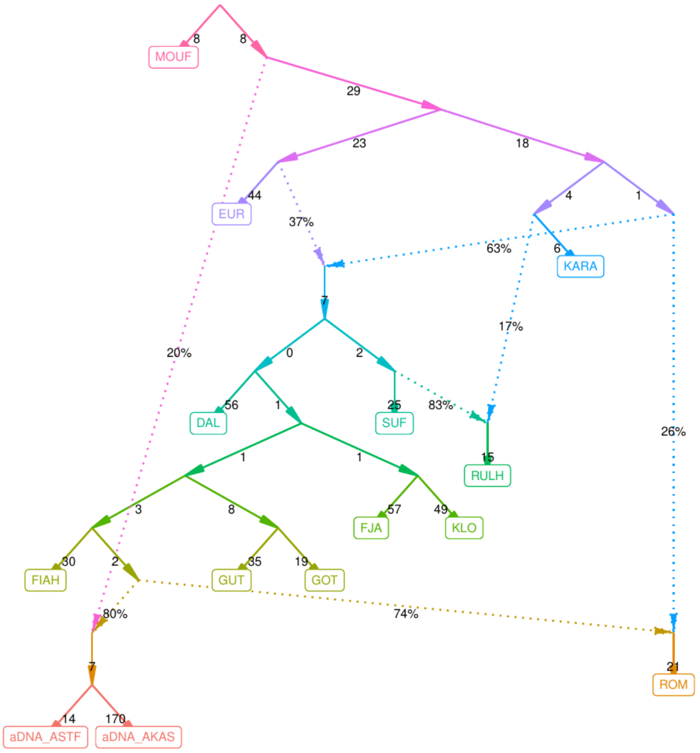

Supplementary Figure 10e: Best fitting admixture graph for m=4 and without constraints.

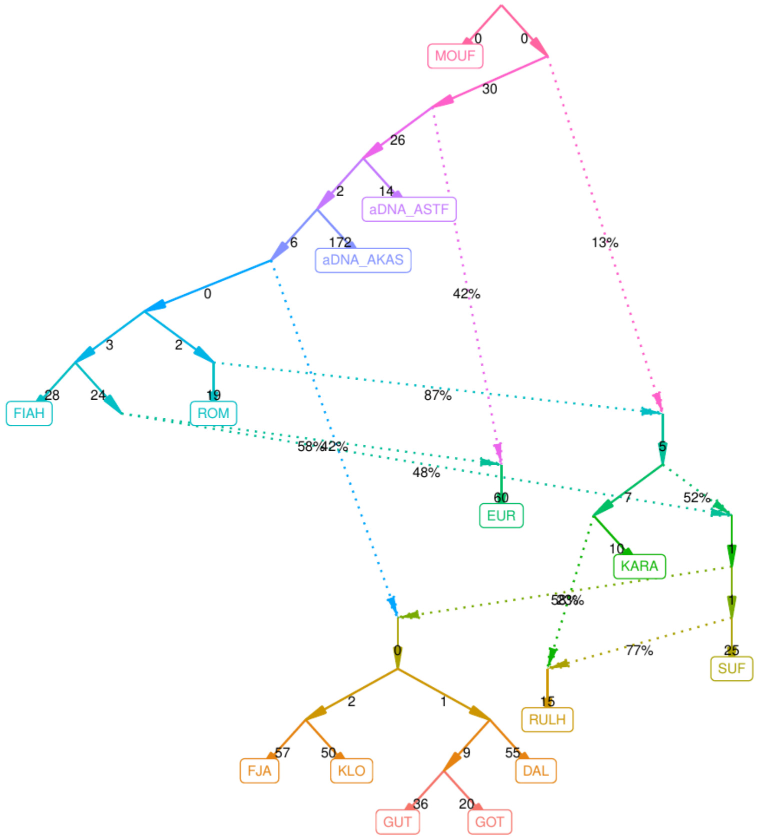

Supplementary Figure 10f: Best fitting admixture graph for m=5 and without constraints.

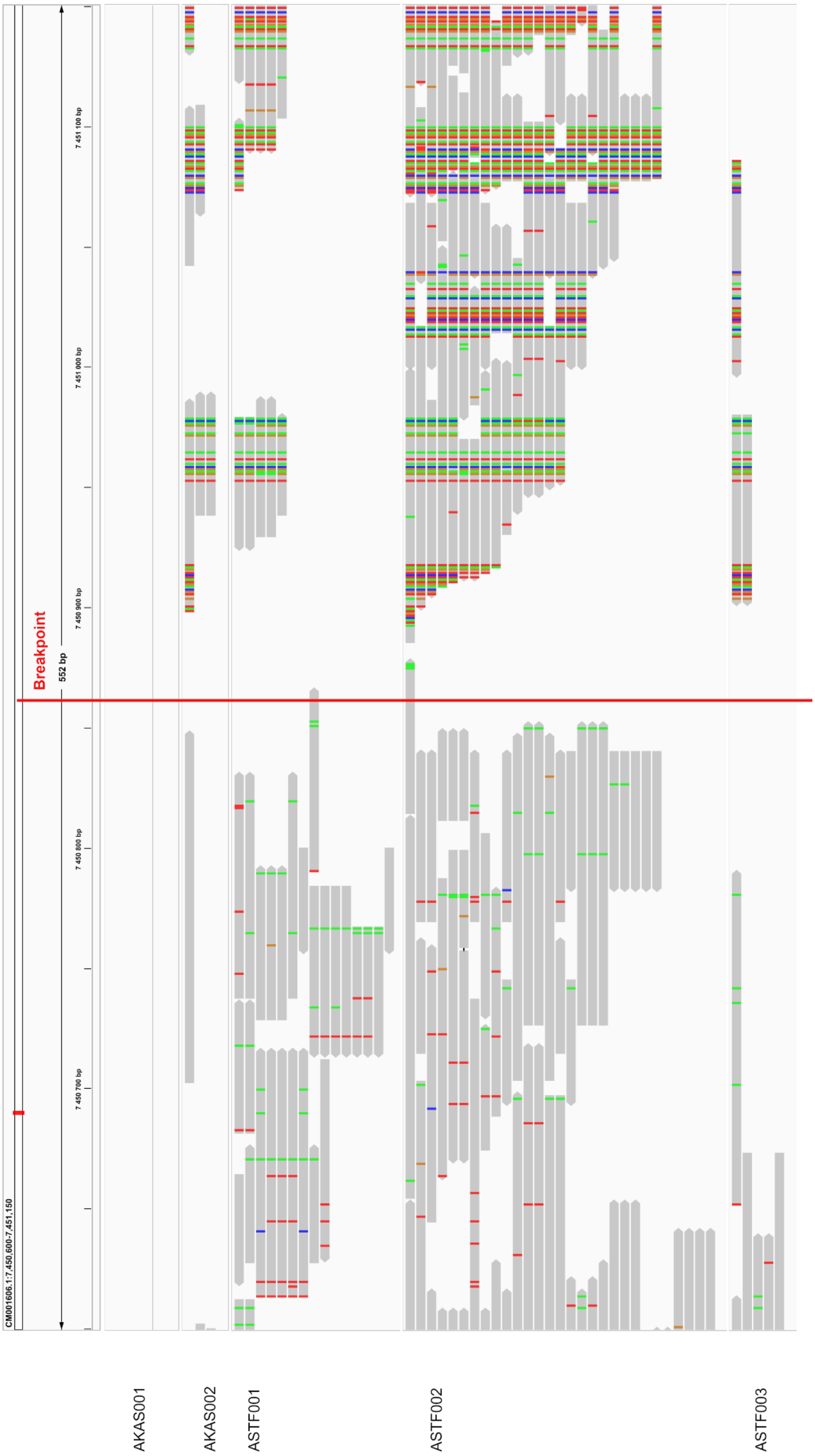

Supplementary Figure 11a: IGV (Robinson et al. 2011) plot of reads mapping to the ancestral ('hairy') reference genome version without the antisense *EIF2S2* retrogene into the

3’ untranslated region (UTR) of the gene *IRF2BP2*. The insertion point is marked by a red line.

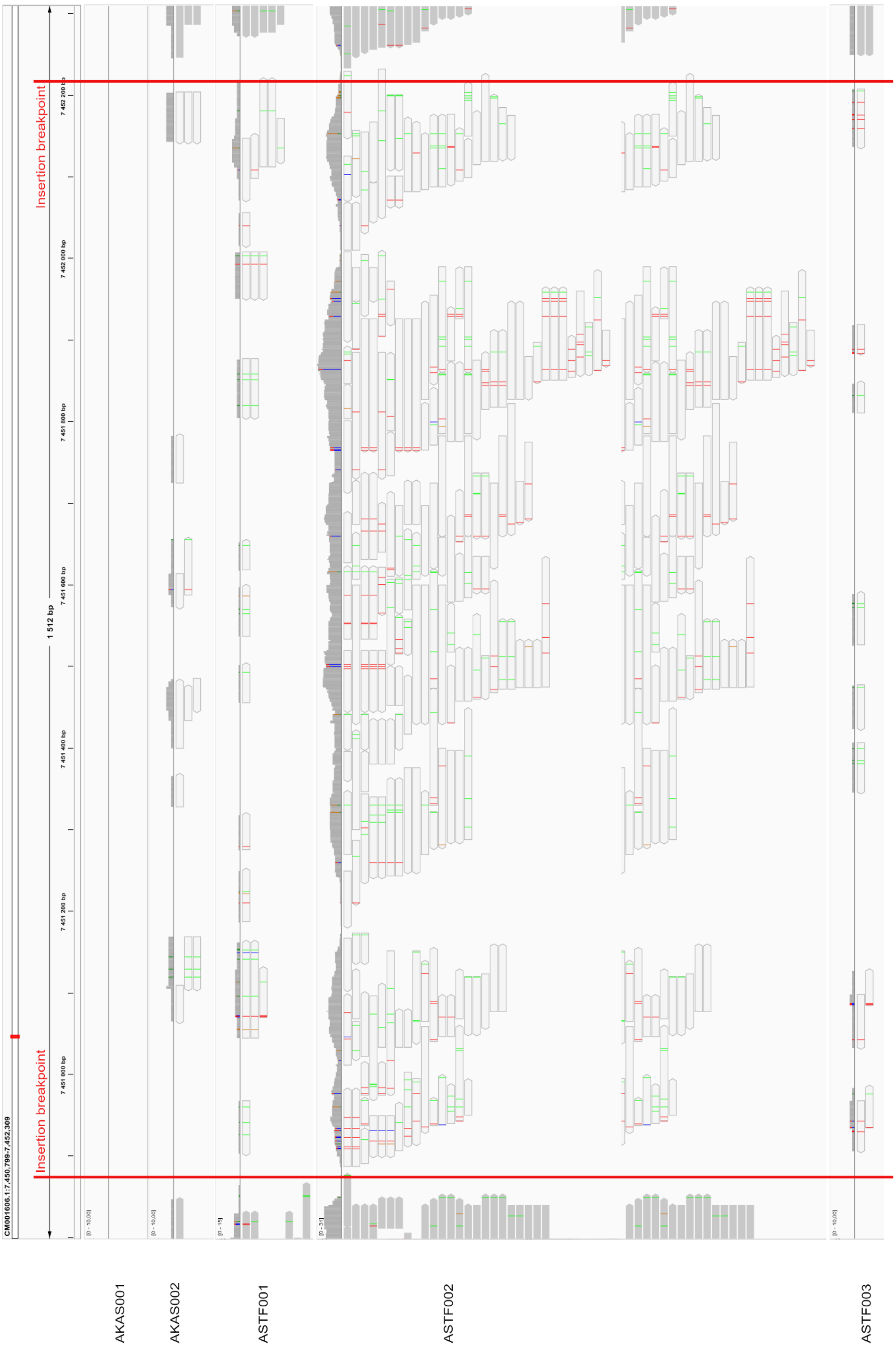

Supplementary Figure 11b: IGV plot (Robinson et al. 2011) of reads mapping to the derived (‘wooly’) reference genome version without the antisense *EIF2S2* retrogene into the 3’ untranslated region (UTR) of the gene *IRF2BP2*. The insertion break points are marked by

red lines. Reads shown in white have a mapping quality of 0, i.e. they map to multiple positions in the genome.

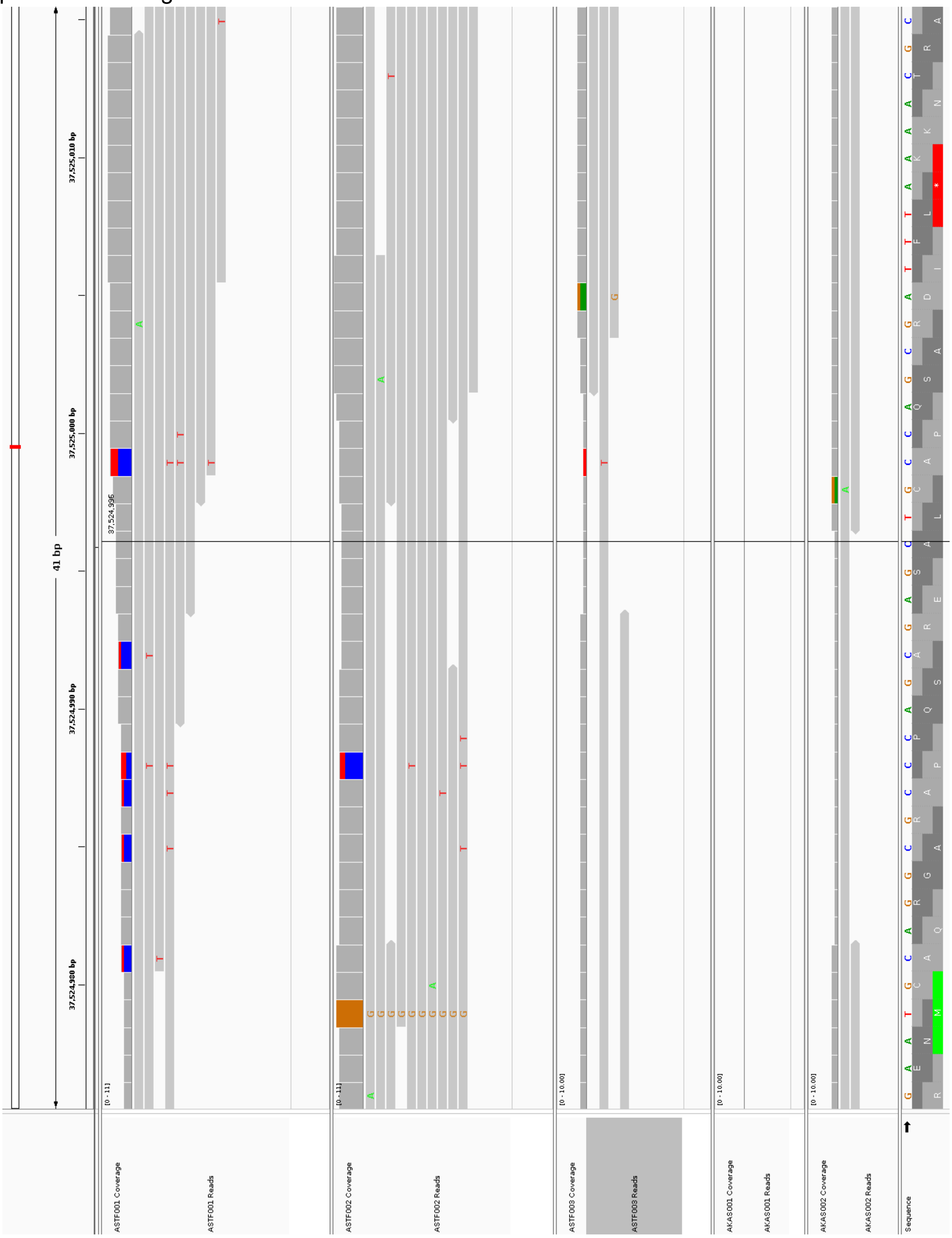

Supplementary Figure 12: IGV plot (Robinson et al. 2011) of reads mapping to the tail length insertion in the 5'UTR of HOXB13 (Lagler et al. 2022) in the ARS-UI_Ramb_v2.0 assembly (Davenport et al. 2022). The black line indicates the insertion breakpoint, absence of the insertion represents the ancestral, i.e. short tail allele.

Additional References in the Supplementary Information

Davenport KM, Bickhart DM, Worley K, Murali SC, Salavati M, Clark EL, Cockett NE, Heaton MP, Smith TPL, Murdoch BM, et al. 2022. An improved ovine reference genome assembly to facilitate in-depth functional annotation of the sheep genome. *GigaScience* 11:giab096.

Lagler DK, Hannemann E, Eck K, Klawatsch J, Seichter D, Russ I, Mendel C, Lühken G, Krebs S, Blum H, et al. 2022. Fine-mapping and identification of candidate causal genes for tail length in the Merinolandschaf breed. *Commun Biol* 5:1–13.

Robinson JT, Thorvaldsdóttir H, Winckler W, Guttman M, Lander ES, Getz G, Mesirov JP. 2011. Integrative genomics viewer. *Nature biotechnology* 29:24–26.
